# Multiomic analysis implicates nuclear hormone receptor signalling in clustering epilepsy

**DOI:** 10.1038/s41398-024-02783-5

**Published:** 2024-01-27

**Authors:** Rebekah de Nys, Clare L. van Eyk, Tarin Ritchie, Rikke S. Møller, Ingrid E. Scheffer, Carla Marini, Rudrarup Bhattacharjee, Raman Kumar, Jozef Gecz

**Affiliations:** 1 Adelaide Medical School and Robinson Research Institute, The University of Adelaide, Adelaide, SA 5005 Australia; 2 Department of Epilepsy Genetics and Personalized Medicine (member of ERN EpiCARE), Danish Epilepsy Centre, Filadelfia, Dianalund Denmark; 3 Department of Regional Health Research, University of Southern Denmark, Odense, Denmark; 4 Epilepsy Research Centre, University of Melbourne, Austin Health, Heidelberg, VIC 3084 Australia; 5 Department of Paediatrics, University of Melbourne, Parkville, VIC 3052 Australia; 6 Department of Neurology, The Royal Children’s Hospital, Parkville, VIC 3052 Australia; 7 Murdoch Children’s Research Institute, Parkville, VIC 3052 Australia; 8 Child Neurology and Psychiatry Unit Children’s Hospital “G. Salesi” Azienda Ospedaliero-Universitaria delle Marche Ancona, Ancona, Italy; 9 South Australian Health and Medical Research Institute, Adelaide, SA 5000 Australia

**Keywords:** Molecular neuroscience, Medical genetics, Psychiatric disorders

## Abstract

Clustering Epilepsy (CE) is an epileptic disorder with neurological comorbidities caused by heterozygous variants of the X chromosome gene *Protocadherin 19* (*PCDH19)*. Recent studies have implicated dysregulation of the Nuclear Hormone Receptor (NHR) pathway in CE pathogenesis. To obtain a comprehensive overview of the impact and mechanisms of loss of PCDH19 function in CE pathogenesis, we have performed epigenomic, transcriptomic and proteomic analysis of CE relevant models. Our studies identified differential regulation and expression of Androgen Receptor (AR) and its targets in CE patient skin fibroblasts. Furthermore, our cell culture assays revealed the repression of *PCDH19* expression mediated through ERα and the co-regulator FOXA1. We also identified a protein-protein interaction between PCDH19 and AR, expanding upon the intrinsic link between PCDH19 and the NHR pathway. Together, these results point to a novel mechanism of NHR signaling in the pathogenesis of CE that can be explored for potential therapeutic options.

## Introduction

*Protocadherin 19 (PCDH19)* pathogenic variants cause the infantile encephalopathy Clustering Epilepsy (CE, previously known as Girls Clustering Epilepsy; GCE, Female-Limited Epilepsy; FE and Epilepsy and Mental Retardation Limited to Females; EFMR: OMIM #300088) [1]. Seizures of this disorder are often febrile, occur in clusters with onset after mini-puberty (8–10 months of age) and generally offset by or during puberty [1–3]. Individuals with CE also present a range of psychiatric comorbidities such as mild to severe intellectual disability (ID), autism spectrum disorder (ASD), hyperactive and/or attention-deficit disorder (ADHD) and late-onset schizophrenia [4]. Variants in the X chromosome gene *PCDH19* cause CE in females and males with heterozygous variants or postzygotic somatic variants respectively, while males with hemizygous variants are asymptomatic carriers [1, 5, 6]. This unique pattern of inheritance and, consequently, the disease mechanism can be best explained by the ‘cellular interference’ model postulating that X-inactivation in CE females and mosaicism in CE males results in altered networks and communications between the PCDH19-Wildtype (WT) and Mutant (MT) expressing neurons, eventually leading to seizures and CE comorbidities [1]. This model is supported by studies in *PCDH19*^*WT/MT*^ female mice that show altered cell sorting between WT and MT expressing cells in the developing cortex and altered mossy fibre presynaptic development due to mismatching between PCDH19-MT and the cell adhesion molecule N-cadherin [7, 8]. However, further systematic studies into the molecular nature of the altered communications between PCDH19-WT and MT cells are required.

PCDH19 is a moonlighting protein with roles in cell adhesion and γ-aminobutyric acid type A receptor (GABA_A_R) binding [9–13]. PCDH19 has also recently been shown to have nuclear function and roles in lysine-specific demethylase 1 (LSD1) and Nuclear Hormone Receptor (NHR) mediated gene regulation [14, 15]. The C-terminal region of PCDH19 has been shown to undergo NMDA receptor (NMDAR)-dependent cleavage by ADAM10 and possibly γ-secretase. The cleaved PCDH19 peptide translocates to the nucleus where it binds to LSD1 to directly regulate the expression of immediate-early genes (IEGs) [15]. In neurons, the activity of LSD1 depends on its splicing by the nuclear protein NOVA1 to generate neuroLSD1 [15]. The NOVA1-mediated splicing of LSD1 has been shown to be regulated by PCDH19 [15]. The NHR pathway is regulated by steroids that can bind and activate their NHRs [such as Oestrogen Receptor (ER) α, Progesterone Receptor (PGR) and Androgen Receptor (AR)] to regulate gene expression via the genomic and non-genomic pathways [2]. Furthermore, neurosteroids can positively and negatively regulate neuron excitability by binding to the transmembrane binding sites of the GABA_A_R. Three lines of evidence have implicated PCDH19 in NHR-mediated gene regulation [16]. Firstly, perturbed steroidogenesis and NHR-related gene expression has been identified in CE affected individuals [17, 18]. Secondly, PCDH19 interacts with the nuclear protein NONO/p54nrb to positively regulate Oestrogen Receptor (ER) α mediated transcriptional activity. Interestingly, PCDH19 CE pathogenic variants are unable to enhance ERα activity [14]. Finally, CE seizure onset during infancy and offset during adolescence has been shown to correlate with natural fluctuations in steroid levels during development [2]. Taken together, these results point to a role for PCDH19 in the regulation of NHR signalling and has led to the inclusion of CE-affected individuals in ganaxolone (a synthetic analogue of the steroid allopregnanolone) clinical trials [19, 20].

To expand our understanding of the role of *PCDH19* variants in CE pathogenesis, we performed CE methylome, transcriptome and proteome studies in diverse cellular models of CE. Our multidimensional analyses implicate PCDH19 in the NHR pathways at the level of methylation, gene regulation and protein-protein interaction and suggests that this pathway plays a critical role in CE pathology.

## Results

### Epigenomic and transcriptomic profiling reveals dysregulation of the steroid pathway

**Fig. 1:**
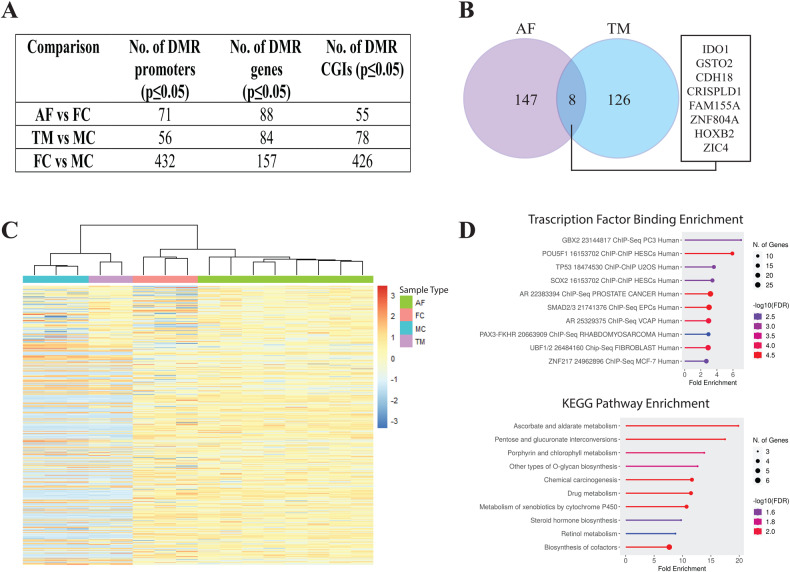
DNA methylation analysis of CE skin fibroblasts. **A** Comparison of the number of DMRs in each group. **B** Venn diagram showing the overlap of genes with differentially methylated promoter and/or gene body in AFs vs FCs and TMs vs MCs comparisons. **C** Hierarchical clustering of samples by DMPs identified in AFs vs. FCs. **D** KEGG pathway and TF binding enrichment analysis of the genes with differentially methylated promoter/gene body identified in AFs (ShinyGO, TF.Target.ChEA.2016 database- FDR cut-off 0.1) [47]. Top 10 most enriched pathways are shown. AF affected female, FC female control, TM transmitting male, MC male control, TF transcription factor.

We explored the gene expression and regulatory mechanisms of CE pathogenesis in patient-derived tissues by performing epigenomic (EPIC-array) and transcriptomic (RNA-sequencing) profiling on skin fibroblasts from affected CE females (AF, *n* = 8, mean age at sampling = 19.2 years) and female controls (FC, *n* = 3, mean age at sampling = 15.7 years) (Supplementary Table 1). We also performed EPIC-array and RNA-sequencing of unaffected transmitting males (TM, *n* = 2, mean age at sampling = 47.5 years) and male controls (MC, *n* = 3, mean age at sampling = 18 years) to determine the effect of pathogenic *PCDH19* variant on the epigenome and transcriptome of hemizygous individuals who are typically unaffected. When comparing affected females vs. female controls, we identified differentially methylated regions (DMRs) in 71 promoters, 88 gene bodies and 55 CpG islands (CGI). Transmitting males vs. male controls identified DMRs in 56 promoters, 84 gene bodies and 78 CGIs (Fig. 1A and Supplementary Table 2 and 3). This indicates significant genome-wide differential methylation in affected females and transmitting males. Of these genes, eight were differentially methylated in both AFs and TMs (*IDO1, GSTO2, CDH18, CRISPLD1, FAM155A, ZNF804A, HOXB2* and *ZIC4*) (Fig. 1B). Hierarchical clustering by differentially methylated probes (DMPs) from all comparisons (affected females vs. female controls, transmitting males vs. male controls and male controls vs. female controls) showed that affected females cluster away from female controls (Fig. 1C). We performed enrichment analysis for transcription factor binding sites (TFs) in the promoters of genes harbouring DMRs for affected females vs. female controls. The data showed enrichment of genes regulated by the NHR Androgen Receptor (AR) as well as the transcription factors GBX2, POUF1, TP53, SOX2, SMAD23, PAX3-FKHR, UBF1/2 and ZNF217 (Fig. 1D). KEGG pathway analysis showed enrichment for processes such as steroid hormone and cofactor biosynthesis (Fig. 1D). Overall, these results show impaired genome-wide AR pathway methylation, a NHR not previously implicated in CE.

**Fig. 2:**
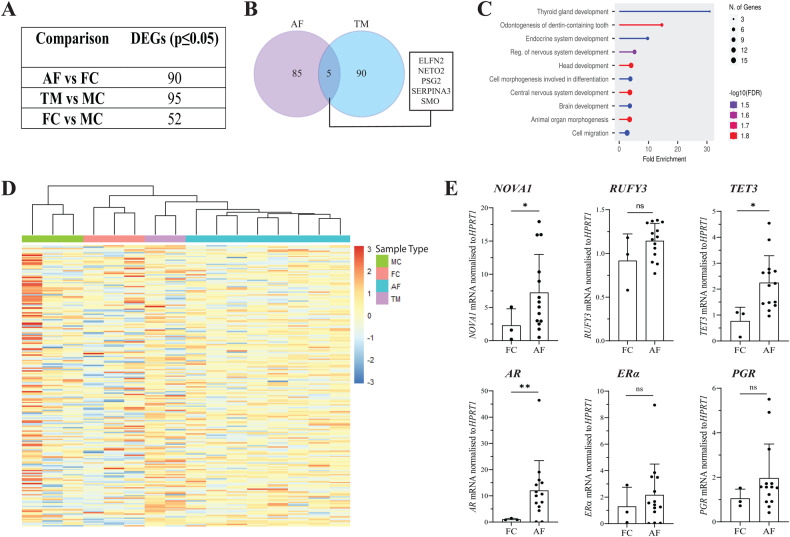
Gene expression analysis of CE skin fibroblasts. **A** Comparison of the number of DEGs in each group. **B** Venn diagram showing the overlap in dysregulated genes when comparing AFs vs. FCs and TMs vs. MCs. **C** Hierarchical clustering of samples by DEGs for AFs vs. FCs. **D** Biological Process enrichment analysis of AF vs. FC DEGs (ShinyGO, Biological Process, FDR cut-off 0.05) [47]. **E** Validation of altered expression of *NOVA1, RUFY3, TET3, AR, ERα* and *PGR* expression in FC (*n* = 3) and AF (*n* = 14) skin fibroblasts by RT-qPCR. Statistical analysis was performed using unpaired *t*-test with Welch’s correction. *p ≤ 0.05, **p ≤ 0.01. AF affected female, FC female control, TM transmitting male, MC male control.

To compare methylation and gene expression differences, we performed RNA-sequencing on RNA extracted from the same patient skin fibroblasts that we used for epigenomic profiling. We identified 90 differentially expressed genes (DEGs) when comparing affected females vs. female controls (56 upregulated and 34 downregulated) and 95 in transmitting males vs. male controls (45 upregulated and 50 downregulated) (Fig. 2A, Supplementary Fig. 1, Supplementary Table 4). Interestingly, hierarchical clustering of DEGs from all comparisons (comparing affected females vs. female controls, transmitting males vs. male controls and male controls vs. female controls) showed that transmitting males clustered with affected females rather than male controls (Fig. 2C). Five genes (*ELFN2, NETO2, PSG2, SERPINA3* and *SMO*) were dysregulated in both the affected female and transmitting male groups (Fig. 2B). Gene ontology analysis of DEGs from affected females vs. female controls showed enrichment for biological processes such as thyroid gland development, brain development and cell migration (Fig. 2D). Our previous microarray studies on affected and control female skin fibroblasts showed an enrichment of dysregulated PGR and ERα targets [17]. However, TF binding enrichment analysis for our current cohort was not able to replicate this finding. We validated dysregulated genes with potential CE relevance by RT-qPCR in affected (*n* = 14) and control female (*n* = 3) skin fibroblasts. These genes were selected based on their role in DNA demethylation (*TET3*), the establishment of neuron polarity (*RUFY3*), PCDH19-regulated RNA splicing (*NOVA1*) and NHR signalling (*AR*) [21–24] (Fig. 2E). As *AR* was significantly upregulated in affected females, we performed RT-qPCR of *ERα* and *PGR* to determine if other NHRs are also dysregulated in affected females. *ERα* and *PGR* showed a trend of higher expression in affected females when compared to female controls, though not statistically significant (Fig. 2E). These data are the first to show dysregulation of *AR* in CE patients.

Promoter methylation can lead to decreased gene expression through blocking transcription factor binding. However, gene body methylation has been linked to increased gene expression through mechanisms that are poorly understood [25]. We identified four genes (*RUFY3, HOXB3, SERPINA3* and *LHX8*) that showed both significant differential methylation and expression in affected females (Supplementary Fig. 2A). To investigate the impact of differential methylation on the expression of these four genes as well as the upregulated methylcytosine dioxygenase *TET3*, we compared gene expression with promoter or gene body methylation. As expected, the expression of *RUFY3* and *HOXB3* negatively correlated with promoter methylation, with affected females having higher expression and lower promoter methylation than female controls (Supplementary Fig. 2B and 2C). Conversely, the expression of *SERPINA3, LHX8* and *TET3* positively correlated with gene body methylation, with affected females having lower expression and higher gene body methylation than female controls (Supplementary Fig. 2C–F). Therefore, altered promoter and gene body methylation may impact the expression of certain genes in affected females.

### *PCDH19* regulation by ERα and its coregulator FOXA1

#### PCDH19 expression is regulated by steroid hormones

**Fig. 3:**
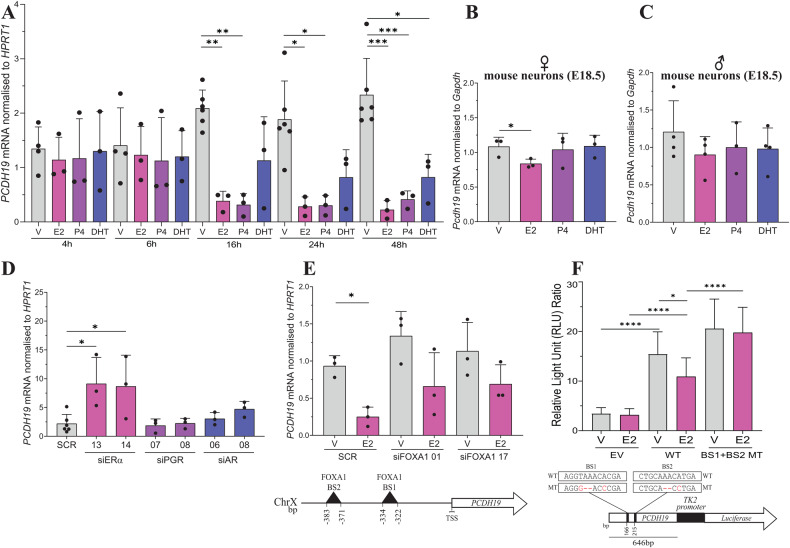
*PCDH19* expression is regulated by steroid treatment. **A** T47D cells were cultured in hormone-depleted conditions for 48 h and then treated with 10 nM E2, 10 nM P4, 10 nM DHT or vehicle (V) for 4, 6, 16, 24 and 48 h. *PCDH19* expression was assayed by RT-qPCR. *n* ≥ 3 biological replicates. Statistical analysis was performed using one-way ANOVA for each timepoint. **B** *Pcdh19* mRNA expression in female (*n* = 3) or **C** male (*n* $\ge$ 3) E18.5 mouse cortical neurons treated with 10 nM E2, 10 nM P4, 10 nM DHT or vehicle (V) for 24 h. Statistical analysis was performed using one-way ANOVA. **D** ERα, PGR and AR were KD in T47D cells with two siRNA molecules and *PCDH19* expression was determined by RT-qPCR. *n* $\ge$ 3. Statistical analysis performed using one-way ANOVA comparing against SCR. **E** FOXA1 was KD in T47D cells with two siRNA molecules and treated with 10 nM E2 or vehicle. *PCDH19* expression was determined by RT-qPCR. *n* = 3. Statistical analysis performed using one-way ANOVA comparing against SCR V. Schematic diagram of the location of two FOXA1 binding sites in the PCDH19 promoter is also shown. **F** MCF-7 cells were transfected with pGL2-TK2 (EV), pGL2-TK2-BS1 + BS2 WT or pGL2-TK2-BS1 + BS2 MT reporter constructs and treated with 10 nM E2 or vehicle. Firefly Luciferase values were normalized to *Renilla* Luciferase transfection control and expressed as Relative Light Units (RLU). Each experiment was performed in four technical and three biological replicates. Statistical analysis was performed using one-way ANOVA. *p ≤ 0.05, **p ≤ 0.01, ***p ≤ 0.001, ****p ≤ 0.0001.

As we identified altered expression and methylation of genes involved in the steroid pathway in our patient cohort, we considered if *PCDH19* itself could be regulated by steroids. To determine the effect of NHRs on *PCDH19* expression we screened a set of breast cancer cell lines to identify one that expresses ERα, PGR and AR. We identified the T47D cell line as expressing the three NHRs, which was used as a model for further studies (Supplementary Fig. 3). To determine the impact of steroid treatment on *PCDH19* expression, we cultured T47D cells in hormone stripped conditions before treating the cells with β-oestradiol (E2), progesterone (P4) and dihydrotestosterone (DHT) for 4, 6, 16, 24 and 48 h. Western blot analysis of ERα, PGR and AR showed expected changes to their protein levels in the cells treated with cognate ligands: that is, the degradation of ERα and PGR in E2 and P4 treated cells, and accumulation of AR in DHT treated cells (Supplementary Fig. 4) The expression of known ERα, PGR and AR target genes confirmed successful ligand-mediated activation of these NHRs (Supplementary Fig. 5). We observed a significant decrease in *PCDH19* expression in cells treated with E2 and P4 treatment for 16, 24 and 48 h, and DHT after 48 h. No change in *PCDH19* expression was observed after 4 and 6 h of steroid treatment (Fig. 3A). Interestingly, we observed an increase in *PCDH19* expression with increasing amount of time cells were cultured in hormone-stripped medium (Fig. 3A). As steroid treatment resulted in the repression of *PCDH19*, we hypothesised that this increased expression could be due to de-repression of *PCDH19* in cells exposed to prolonged hormone-depleted culture conditions. We tested this observation by assaying the *PCDH19* expression in T47D cells cultured in stripped and non-stripped (normal) conditions for 52 and 72 h. Consistent with our prediction, we found that prolonged cell culture in stripped conditions results in an increase in *PCDH19* expression (Supplementary Fig. 6). Together, this data suggests that *PCDH19* is repressed by steroids.

To determine if *Pcdh19* is repressed by steroid treatment in disease relevant cells, we cultured primary mouse E18.5 female and male cortical neurons with E2, P4, DHT or vehicle for 24 h. The ligand-mediated activation of known ER${\rm{\alpha }}$, PGR and AR target genes was confirmed by RT-qPCR (Supplementary Fig. 7 and 8). *Pcdh19* was significantly repressed by E2, but not P4 or DHT in female neurons (Fig. 3B). However, *Pcdh19* expression was not significantly repressed in E2, P4 or DHT treated male neurons (Fig. 3C). These results suggest that E2-mediated *PCDH19* repression may be female-specific, occurring across species and tissue types.

### *PCDH19* expression is repressed by ERα

NHR-mediated gene regulation is complex and can depend on the presence, interaction and activation of multiple NHRs [26]. To determine how different and multiple NHR activation affects *PCDH19* expression, we measured *PCDH19* mRNA levels in T47D cells treated with equal concentrations of two or more of the steroids E2, P4 and DHT. *PCDH19* expression was reduced in E2 + P4, E2 + DHT and E2 + P4 + DHT but not DHT + P4 treated cells (Supplementary Fig. 9), suggesting that the presence of E2 is required for *PCDH19* repression in a background of multiple steroids.

Given that E2 is the major ligand for ERα we set out to confirm if ERα is responsible for *PCDH19* repression. We knocked down (KD) ERα, PGR or AR using two individual siRNAs against each target. Successful knockdown was confirmed by western blotting (Supplementary Fig. 10). We observed that ERα KD results in increased *PCDH19* expression while PGR and AR KD do not change *PCDH19* levels when compared to scrambled siRNA control (Fig. 3D). To identify the minimum required concentration of E2 for *PCDH19* repression, we treated T47D cells with various E2 concentrations and found that *PCDH19* repression occurred in cells treated with as little as 1 nM E2 (Supplementary Fig. 11). Together these results suggest ERα to be the primary NHR involved in *PCDH19* repression.

### *PCDH19* repression by ERα is FOXA1 dependent

*PCDH19* repression in T47D cells occurs after 16 h of E2 treatment (Fig. 3A) and we could not identify any NHR binding sites within the *PCDH19* promoter in publicly available ChIP-seq data. Therefore, we hypothesised that this regulation may not be occurring through direct ERα binding to the *PCDH19* promoter, but instead through another transcription factor. Analysis of publicly available ChIP-seq datasets identified two Forkhead box A1 (FOXA1) binding sites located upstream of the *PCDH19* transcription start site [27] (Fig. 3E). We named these sites BS1 (AGGTAAACACGA) and BS2 (CTGCAAACATGA). FOXA1 is a pioneering transcription factor known to bind to condensed chromatin and enhance the transcriptional regulatory activity of ERα and AR [28]. Over 95% of oestrogen regulated genes require FOXA1 for oestrogen regulation in MCF-7 breast cancer cells [29]. To determine if FOXA1 is required for *PCDH19* regulation, we KD FOXA1 in T47D cells with two independent siRNA molecules and treated the cells with vehicle or E2 (Supplementary Fig. 12). FOXA1 KD reduced the E2-mediated activation of *TFF1*, a known FOXA1-dependent ERα target (Supplementary Fig. 13). FOXA1 KD also abrogated E2-mediated repression of *PCDH19* (Fig. 3E). To confirm that FOXA1 is responsible for ERα-mediated *PCDH19* repression, we generated a firefly luciferase reporter construct by inserting a putative *PCDH19* regulatory fragment carrying the two FOXA1 binding sites upstream of a TK2 promoter in the pGL2-TK2 plasmid. We assayed the luciferase reporter activity of this construct in transfected MCF-7 cells treated with E2 or vehicle for 24 h. Compared to the pGL2-TK2 plasmid, the reporter construct with the *PCDH19* promoter fragment showed increased luciferase activity that was reduced by ~27% in the E2 treated cells (Fig. 3F**)**. To confirm that FOXA1 is responsible for E2-mediated regulation of the *PCDH19* promoter fragment, we mutated the BS1 (AGGG--ACCCGA) and BS2 (CTGCA--CCTGA) sites to eliminate FOXA1 binding. Our results showed that mutation of both BS1 + BS2 abolished E2-mediated repression of luciferase activity, suggesting that the two FOXA1 binding sites are important for *PCDH19* regulation (Fig. 3F). Overall, our results showed that ERα-dependent repression of *PCDH19* is mediated by FOXA1 through its binding to the *PCDH19* promoter.

### PCDH10, PCDH12 and PCDH19 interact with AR

**Fig. 4:**
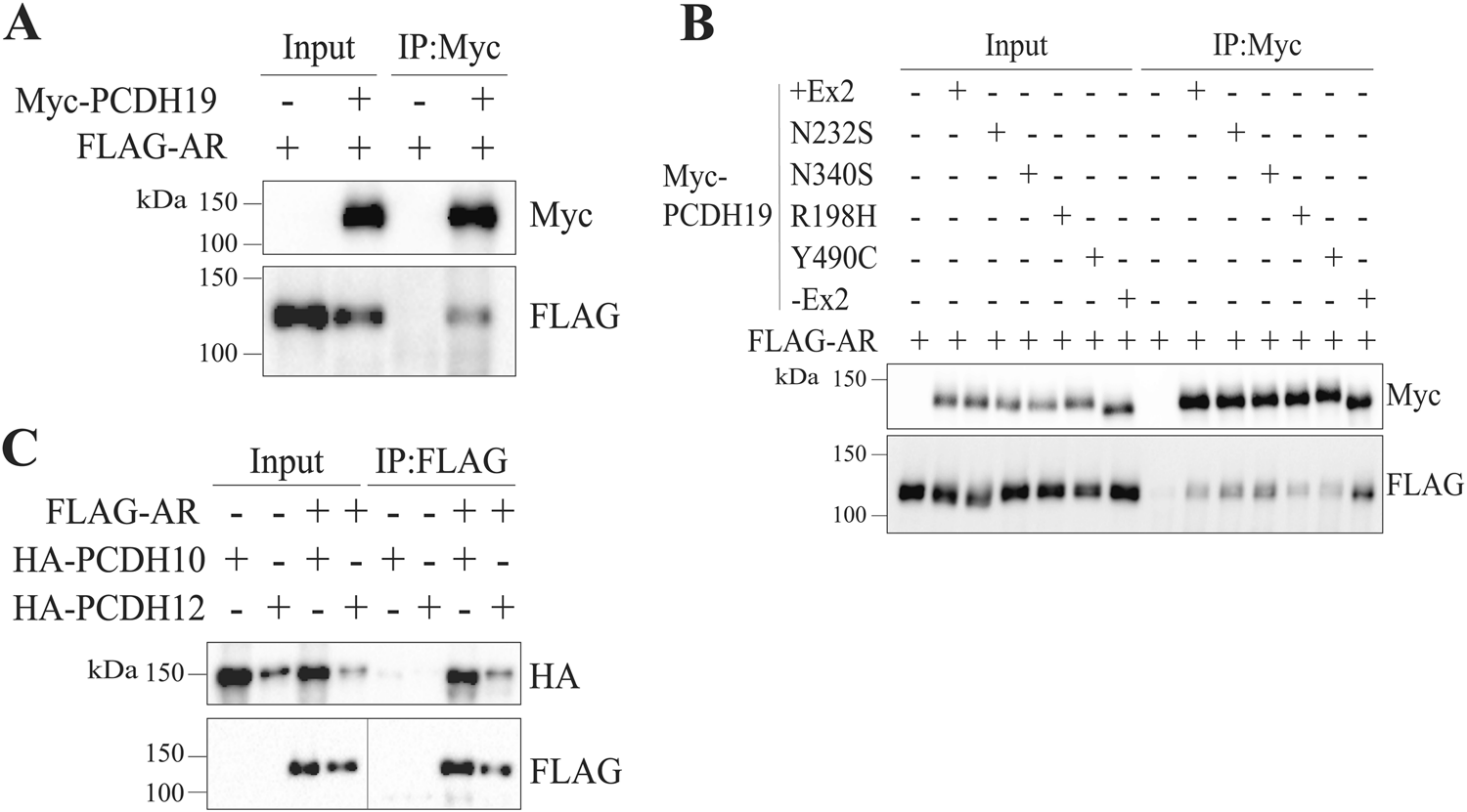
Androgen Receptor interacts with PCDH19. **A** Myc-PCDH19 was immunoprecipitated with anti-Myc magnetic beads. Inputs and IP samples were western blotted to detect Myc-PCDH19 and FLAG-AR. **B** Myc-PCDH19 isoforms (+Ex2 or -Ex2) and pathogenic missense variants were immunoprecipitated with anti-Myc magnetic beads. Inputs and IP samples were western blotted to detect Myc-PCDH19 and FLAG-AR. **C** FLAG-AR was immunoprecipitated with anti-FLAG agarose beads. Inputs and IP samples were western blotted to detect FLAG-AR, HA-PCDH10 and HA-PCDH12.

Our previous studies found that PCDH19 with NONO positively regulates ERα transcriptional activity, with PCDH19 WT but not CE pathogenic variant protein enhancing ERα transcriptional activity [14]. Considering the functional association of PCDH19 with ERα, we asked if PCDH19 interacts with one or more of these NHRs. To investigate this, we performed co-immunoprecipitation (co-IP) using protein lysates of HEK293T cells expressing NHRs with FLAG tag (FLAG-ERα, FLAG-PGR or FLAG-AR) alone or with Myc-tagged PCDH19 (Myc-PCDH19). Although we could not detect a PCDH19-PGR or PCDH19-ERα interaction (*data not shown*), we did observe a PCDH19-AR interaction (Fig. 4A). PCDH19 cell adhesion and gene regulatory function has been shown to be affected by *PCDH19* pathogenic variants [12, 14, 30]. Furthermore, PCDH19 has two major isoforms resulting from the alternative splicing of exon 2: PCDH19 + Ex2 and PCDH19-Ex2 [5]. As the PCDH19 nuclear localisation signal spans exons 2 and 3 [14, 15], alternative splicing of exon 2 may lead to different functions for PCDH19 + Ex2 and PCDH19-Ex2. To determine if the AR-PCDH19 interaction is ablated by PCDH19 variants or isoform, we performed co-IP of HEK293T cell lysates expressing pathogenic PCDH19 variants or PCDH19-Ex2 isoform alone or with FLAG-AR. Our results show that the AR-PCDH19 interaction is not disrupted by PCDH19 variant or isoform type (Fig. 4B).

PCDH19 belongs to the δ2 subclass of the non-clustered protocadherin family that also includes the protocadherins (PCDHs) 10, 17 and 18 [31]. PCDH12 is structurally similar to the other δ2 protocadherins, albeit it lacks the CM1 and CM2 domains [31]. To determine if the AR-PCDH19 interaction also occurs with other δ2-PCDHs, we performed co-IP of protein lysates of HEK293T cells expressing HA-tagged PCDH10 or PCDH12 (HA-PCDH10 or HA-PCDH12) alone or with FLAG-AR. Interestingly, AR co-IPed with both PCDH10 and PCDH12, demonstrating that AR interacts with other δ2-PCDHs (Fig. 4C). Overall, these are the first results showing PCDH19 (and other PCDHs) interaction with AR, further linking PCDH19 to the NHR pathway.

## Discussion

CE is a complex childhood-onset (median age 10 months) epilepsy syndrome characterized by clusters of febrile and afebrile seizures, developmental delay or cognitive impairment, features of autism spectrum disorder, and behavioural abnormalities including ADHD [1, 4]. Since the identification of the *PCDH19* gene as the genetic cause of CE, the research community has generated a considerable amount of data delving into its molecular pathogenesis. Among these are disordered steroidogenesis and NHR-mediated gene dysregulation in CE [14, 17, 18]. The data presented here provide fundamental experimental evidence supporting these findings, while also pointing to new pathways potentially involved in CE. One such finding is the upregulation of *NOVA1* expression in the skin fibroblasts of affected compared to control females. Importantly, NOVA1 is known to generate the neuron-specific isoform of LSD1 (neuroLSD1), a known interactor of PCDH19, which regulates the expression of immediate early genes (IEGs). A study by Gerosa et al. (2022) found that *PCDH19* downregulation increases the expression of neuroLSD1 and NOVA1, thereby affecting downstream expression of IEGs [15]. Our results corroborate the findings by Gerosa et al. (2022) and show that CE patients have significantly upregulated *NOVA1*. Another interesting finding from our multi-omics investigation was the upregulation of genes involved in DNA methylation maintenance, specifically the overexpression and gene body hypermethylation of *TET3* in CE females. TET3 is a member of the Translocation methyl-cytosine dioxygenase (TET) family of proteins (which includes TET1 and TET2) and is involved in initiating DNA demethylation [32]. *TET3* is highly expressed in the cortex and hippocampus and is implicated in the regulation of genes involved in memory, neuronal activity and synaptic plasticity [33]. Previous studies have found that neuronal *TET3* expression is regulated by synaptic activity and that TET3 can regulate surface Glutamate receptor 1 (GluR1) levels to influence synaptic transmission [34]. Therefore, upregulation of *TET3* could have a major impact not only on methylation and subsequent gene expression in CE individuals, but also synaptic transmission. Overall, our transcriptomic and epigenomic studies provide evidence of *NOVA1* and *TET3* involvement in CE pathogenesis.

Our epigenomic, transcriptomic and proteomic results have implicated the NHRs in CE and indicate novel roles of ERα and AR in CE. Our RNA-sequencing, epigenomic and proteomic results showed an intrinsic link between PCDH19 and AR. CE fibroblasts show upregulation of *AR* and altered methylation of AR genomic targets and PCDH19 interacts with AR in HEK293T cells expressing epitope-tagged PCDH19 and AR proteins. Previous microarray studies in patient skin fibroblasts [17] and mouse differentiated *Pcdh19* WT and KO neural stem/progenitor cells (mNSPCs) [35] show dysregulation of the ERα signalling pathway. This dysregulation can be explained by the known role of PCDH19 in the promotion of ERα transcriptional activity, while PCDH19 CE-variants fail to enhance ERα transcriptional activity [14, 30]. However, the molecular explanation for the impact of *PCDH19* CE-variants on *AR* and the expression/methylation of its genomic targets is unknown. One possibility is that PCDH19 is a co-regulator of AR transcriptional activity - as it is for ERα – a possibility that was not explored in this study. It should be noted that ERα and AR are known to be linked through direct interaction, expression regulation by oestrogenic and androgenic metabolites, and transcriptional crosstalk [26, 36–38]. Though AR is generally considered to have an antagonistic effect on ERα by occupying subsets of Oestrogen Response Elements, the transcriptional outcome of ERα/AR crosstalk is now known to be highly context dependent [26]. Therefore, the functional interaction between ERα and AR, and any potential role of PCDH19, is a complex issue that requires further study.

The implications of the NHR pathway in CE pathology is strengthened by the finding that *PCDH19* is regulated by oestradiol treatment. We show that *PCDH19* expression itself is regulated by oestrogen via its receptor ERα and coregulator FOXA1 in both T47D cells and female embryonic mouse primary neurons. Interestingly, female-specific ERα regulatory affects are consistent with the observation that treatment of male mouse cortical neurons with E2 did not significantly repress *Pcdh19*. This result may be explained by sex differences of NHR expression or female-specific NHR regulation of gene expression involving PCDH19 [39]. A recent study by Gengenhuber et al. (2022) investigated the neuronal targets of ERα and their effect on brain sex differentiation. They found that treatment of female mice with oestrogen at birth results in a decrease in *Pcdh19* expression in Esr1+ neurons in the posterior bed nucleus of the stria terminalis (BNSTp), though not reaching statistical significance. Interestingly, expression of many other protocadherins were found to increase (*Pcdh7, Pcdh9, Pcdh10, Pcdh15* and *Pcdh11x*) or decrease (*Pcdh1, Pcdh8, Pcdh18* and *Pchd20*) with oestrogen treatment [39]. Other studies in female (but not male) rats and MCF-7 cells found altered expression of protocadherins (including *PCDH19*) on oestrogen and oestrogen-mimics treatment [40, 41]. Taken together, the results strongly suggest that ERα-mediated gene regulation may extend to other protocadherins and the effect of oestrogen treatment on gene expression may differ depending on sex.

The connection of the NHR pathways to CE could pave the way for developing novel anti-seizure medication for this disorder. The identification of altered steroidogenesis and NHR target gene expression in CE patients has led to the inclusion of CE patients in clinical trials for ganaxolone [19, 20]. Other neurosteroids, such as oestrogen and progesterone, have been found to have beneficial effects in various animal and human epilepsy studies [42, 43]. These results are promising, though factors such as sex, age, hormone levels and dosage should be considered before therapeutic administration [44]. In conclusion, our epigenomic, transcriptomic and proteomic results implicate NHR signalling in CE, shedding new light on its pathogenic mechanism and paving the way for exploring NHR-PCDH19 regulated pathways as future potential therapeutic options.

## Materials and methods

### Cell culture

T47D and ZR-75-1 cells and primary skin fibroblasts were maintained in RPMI-1640, GlutaMAX (Gibco) and 10% Heat Inactivated Foetal Bovine Serum (HI-FBS, Gibco). HEK293T (ATCC, 293T-CRL-3216) and MCF-7 (ECACC# 86012803) cells were maintained in DMEM (Gibco) and 10% HI-FBS. T47D and ZR-75-1 cells were kindly provided by the Dame Roma Mitchell Cancer Research Laboratories, the University of Adelaide. All cell lines were mycoplasma free. Primary mouse neurons were maintained in neural-feed media [comprising neurobasal A media (Thermo Fisher, cat #10888-022), 2% B27 (Gibco) and 1% penicillin-streptomycin (Gibco)]. All cells were cultured at 37°C and 5% CO_2_.

### Mouse experimental model

All mouse work was conducted following approval by The University of Adelaide Animal Ethics Committee (AEC No. #395) [45] in accordance with the Australian code for the care and use of animals for scientific purposes. Mouse sample sizes were selected for one-way ANOVA statistical analysis. C57BL/6 mouse strain was used.

### Methylation assay for fibroblast gDNA

DNA methylation for fibroblast gDNA was assessed using the Illumina Infinium MethylationEPIC BeadChip as a service by the Australian Genome Research Facility. Methylation array data was analysed using minfi, limma and mCSEA implemented through the shinyÉPICo package in R v4.1.2 [46]. For DMPs, a cut-off of 20% differential methylation was applied with adj_*P*-value_ < 0.05. For DMR analysis, a cut-off of 10% differential methylation with FDR < 0.01 was applied. For identification of DMRs correlating with changes in gene expression, the mCSEA function mCSEATest was first applied to quantile normalised ranked beta values, then the mCSEAIntegrate function was applied to integrate DMRs with DESeq2 normalised counts from RNAseq (see below, mCSEA v1.12.0, DESeq2 v1.34.0). For DMRs in promoters, only negative correlations were considered biologically relevant, while for DMRs in the gene body, correlation in either direction was considered.

### RNA-sequencing

RNA sequencing was performed as a service by Azenta Biotech. Poly A selected, unstranded libraries were prepared from patient fibroblast total RNA and sequenced on a Novaseq instrument in 2 × 150 bp paired-end (PE) configuration to give a total of 40–60 million reads of raw data per sample. Transcript-level abundances were quantified using Salmon against the pre-built Refgenie hg38 indices (http://refgenomes.databio.org/v3/assets/splash/2230c535660fb4774114bfa966a62f823fdb6d21acf138d4/salmon_sa_index), and aggregated to gene level counts using the tximport function of DESeq2 in R v4.1.2. Differential gene expression analysis was performed using DESeq2 v1.34.0 [46].

### Enrichment analysis

Transcription factor target enrichment analysis of annotated DEGs and DMRs from cultured skin fibroblasts was performed using ShinyGO 0.76.3 (http://bioinformatics.sdstate.edu/go/) [47]. A background list of genes expressed in skin fibroblasts was used for analysis of DEGs identified in patient skin fibroblasts (Supplementary Table 5).

### Mouse E18.5 primary neuron culture

Primary cortical neurons were harvested from E18.5 mouse embryonic brains as previously described [48] and plated in wells precoated with poly-L-lysine in neural-seed media (neurobasal A media, 2% B27, 1% penicillin-streptomycin and 10% HI-FBS) and allowed to adhere for at least 3 h before the medium was changed to neural-feed media [neural-seed media without FBS and 1% GlutaMAX (Gibco)]. The media was half changed every 2–3 days. Investigators were not blinded to the experimental conditions.

### Steroid treatment of T47D cells

5×10^5^ T47D cells were plated in 6-well dishes and cultured in RPMI-1640 and 10% HI-FBS for 24 h. The next day, the cells were washed with 1× PBS and the media was replaced with RPMI-1640 (phenol red free) (Thermo Fisher) and 10% Dextran Coated Charcoal stripped (DCC) FBS for 48 h prior to steroid treatment. The cells were treated with either 10 nM β-oestradiol (E2) (Sigma), 10 nM progesterone (P4) (Sigma), 10 nM dihydrotestosterone (DHT) (Selleck Chemicals) or vehicle (100% EtOH) for the indicated times and then harvested. Cells were stored at −80 °C until analysed.

### Steroid treatment of primary mouse neurons

3.2 × 10^6^ E18.5 mouse cortical neurons were plated in a 6 cm petri dish in neural-seed media. Three days later the media was changed to neural-stripped media (comprising neurobasal A media (phenol red free) (Gibco), 2% B-27, 1% GlutaMAX and 1% penicillin-streptomycin). Three days later the cells were washed with 1× PBS and the media were changed to neurobasal A media with 10% DCC-FBS and cultured for a further 4 days, with the medium half changed every 2 days. The neurons were then treated with 10 nM E2, 10 nM P4, 10 nM DHT or vehicle for 24 h and harvested by scraping for immediate RNA isolation.

### Western blotting

Cell pellets were resuspended in lysis buffer (50 mM Tris-HCl pH 7.5, 150 mM NaCl, 0.2% Triton-X-100, 2 mM EDTA, 0.01% SDS, 50 mM NaF, 0.1 mM Na_3_VO_4_, 1×Protease Inhibitor/no EDTA), sonicated (20% amp for 15 s) and centrifuged at 15000 rpm for 15 min at 4 °C. 10 µg of cell lysate was denatured and resolved by 6% homemade SDS-PAGE protein gel [49]. Proteins were transferred onto nitrocellulose membranes and blocked with 10% skim milk 1× Tris-buffered saline, 0.1% Tween 20 (TBST). The membrane was probed with primary antibody; rabbit anti-AR (Santa Cruz, cat #sc-816), mouse anti-ER${\rm{\alpha }}$ (Santa Cruz, cat #sc-8002), rabbit anti-PGR (Leica Biosystems, # NCL-L-PGR AB), rabbit anti-FOXA1 (Abcam, cat # ab23738), mouse anti-Myc (Sigma Aldrich, cat #M4439), mouse anti-V5 (Thermo Fisher, cat #46-0705), mouse anti-FLAG (Sigma Aldrich, cat #F3165) or rabbit anti-β-Tubulin (Abcam, cat #ab6046) in 1× TBST/2% skim milk overnight at 4°C. The next day, membranes were probed with anti-mouse immunoglobulins/HRP (Dako, cat #P0447) or goat anti-rabbit immunoglobulin/HRP (Dako, cat #P0448) in 1× TBST/2% skim milk, washed with 1× TBST and detected by Clarity Western enhanced chemiluminescence (ECL) substrate (Bio-Rad) using BioRad ChemiDoc MP Imaging System with Imagining Lab 5.0.

### RNA extraction and Reverse Transcription-quantitative PCR (RT-qPCR)

RNA was extracted using QIAshredder (Qiagen) and RNeasy Kit (Qiagen) with on-column RNase-free DNase treatment (Qiagen) according to manufacturer’s protocol. 2 µg of RNA was reverse transcribed using SuperScript IV (Thermo Fisher) and manufacturers protocol. qPCRs for *AR, ERα, PGR, RUFY3, HPRT1, TFF1, PPL, CDKN1A, PCDH19, NOVA1, TET3, Gapdh, Tff1, Rara, Nrip1, Hsd11b2, Ppl, Pip, Cdkn1a, Pmepa1* and *Pcdh19* were performed using TaqMan probes (Thermo Fisher, see Supplementary Table 6) with Taqman Gene Expression Master Mix (2×) (Thermo Fisher) and diluted cDNA. qPCR was performed using the following cycling conditions: hold at 50 °C for 2 min and 95 °C for 10 min, initial denaturation at 95 °C for 10 min followed by 40 cycles of 95 °C for 15 s and 60 °C for 1 min. qPCRs for *GREB1, HSD11B2, PSA* and *HPRT1* were performed using previously published primers (see Supplementary Table 7) with Power SYBR Green Master Mix (Thermo Fisher) and diluted cDNA [17, 50–52]. qPCR was performed using the following cycling conditions: hold at 95 °C for 10 min, 40 cycles of 95 °C for 15 s 60 °C for 1 min, melt curve: 95 °C for 15 s, 60 °C for 30 s and 95 °C for 15 s. Data was acquired using StepOne Real-Time PCR System and software V 2.0 (Applied Biosystems) and expression of the target gene was normalized against *HPRT1* or *Gapdh*.

### siRNA knockdown

3 × 10^5^ cells/well were plated in a 6 well plate. Next day, the cells were transfected with two independent siRNAs for FOXA1, ER${\rm{\alpha }}$, AR or PGR (Dharmacon) or scrambled control (GenePharma) using lipofectamine RNAiMAX reagent (Thermo Fisher). The media was changed to RPMI-1640 and 10% DCC-FBS and cultured at 37 °C/5% CO_2_. The cells were harvested after 36 h. Cells with scrambled or FOXA1 KD were treated with 10 nM E2, 10 nM P4, 10 nM DHT or vehicle for 24 h before harvesting.

### Generation of the FOXA1 binding sites luciferase reporter vector

pGL2-TK2 was purchased from Promega. The sequence of the *PCDH19* promoter and FOXA1 binding sites was obtained from the USCS genome browser (https://genome.ucsc.edu/) with the genomic coordinates chrX:100410177-100410816 of the GRCh38/hg38 assembly. This region was amplified from control female blood gDNA with Platinum SuperFi PCR Master Mix (Thermo Fisher). Amplification was performed using the following conditions: hold at 98 °C 30 s, 38 cycles of 98 °C 15 s, 60.5 °C 15 s, 72 °C 60 s, 72 °C 10 min. PCR mutagenesis of FOXA1 binding sites (BS) 1 and 2 were performed by overlap extension PCR using the same conditions and reagents mentioned above (see Supplementary Table 8 for primer sequences). The amplified *PCDH19* promoter fragment with FOXA1 binding sites was cloned into pGL2-TK2 at *Xho*I and *Xma*I restriction sites.

### FOXA1 binding sites luciferase reporter assay

1.2 × 10^5^ MCF-7 cells/well were plated in a 24 well plate in DMEM (phenol red free) (Sigma Aldrich) and 10% DCC-FBS. The next day cells were transfected with 10 ng pRL-TK and 750 ng pGL2-TK2 or pGL2-TK2-PCDH19-FOXA1 reporter plasmids (WT, BS1 MT, BS2 MT or BS1 + BS2 MT) using Lipofectamine 3000 reagent according to manufacturer’s protocol. 24 h later the cells were treated with 10 nM E2 or vehicle for 24 h and the luciferase assay performed using Dual-Luciferase Reporter Assay System (Promega) and the GloMax 20/20 Luminometer. Firefly luciferase values were normalized to Renilla luciferase values and expressed as Relative Light Units (RLU). All luciferase reporter assays were performed in four technical and three biological replicates.

### Co-immunoprecipitation of epitope tagged proteins

HEK293T cells were plated at 5 × 10^5^ cells/well in 6-well plates. The next day the cells were transfected with various expression plasmids using Lipofectamine 3000 reagent. The cells were harvested 24 h later and lysed by sonication (Sonic’s Vibra-Cell VCX) in IP Buffer. The lysate was clarified by centrifuging at 13000 rpm, 20 min, 4 °C and the supernatants incubated with anti-c-Myc-Magnetic Dynabeads (Thermo Fisher, cat #88843), anti-HA agarose beads (Sigma Aldrich, cat #E6779) or anti-FLAG M2 agarose beads (Sigma Aldrich, cat #A2220) overnight at 4 °C. Next day, the beads were washed three times with IP Buffer and twice with 20 mM Tris-HCl pH 7.5. The proteins were eluted in 40 μL 1× SDS protein-loading buffer (62.5 mM Tris-HCl, pH 6.8, 2% SDS, 10% glycerol, 5% β-mercaptoethanol) for 5 min at 95 °C [49].

### Statistical analysis

Statistical analysis was performed using GraphPad Prism (v8.0). An ordinary one-way analysis of variance (ANOVA) with Dunnet comparison test was used for multiple comparisons with 95% confidence interval. For single comparisons, an unpaired *t*-test with Welch’s correction and 95% confidence interval was used. Bar graphs were plotted showing mean and standard deviation. Statistically significant comparisons are illustrated. Non-significant comparisons (ns) are illustrated where necessary.

### Supplementary information

Supplementary Information

Supplementary Table1

Supplementary Table2

Supplementary Table3

Supplementary Table4

Supplementary Table5

## Data Availability

The data generated or used during this work are available subject to compliance with our obligations under human research ethics from the corresponding author upon reasonable request.

## References

[1] Dibbens LM, Tarpey PS, Hynes K, Bayly MA, Scheffer IE, Smith R (2008). X-linked protocadherin 19 mutations cause female-limited epilepsy and cognitive impairment. Nat Genet.

[2] de Nys R, Kumar R, Gecz J (2021). Protocadherin 19 clustering epilepsy and neurosteroids: opportunities for intervention. Int J Mol Sci.

[3] Liu A, Xu X, Yang X, Jiang Y, Yang Z, Liu X (2017). The clinical spectrum of female epilepsy patients with PCDH19 mutations in a Chinese population. Clin Genet.

[4] Kolc KL, Sadleir LG, Scheffer IE, Ivancevic A, Roberts R, Pham DH (2019). A systematic review and meta-analysis of 271 PCDH19-variant individuals identifies psychiatric comorbidities, and association of seizure onset and disease severity. Mol Psychiatry.

[5] Depienne C, Bouteiller D, Keren B, Cheuret E, Poirier K, Trouillard O (2009). Sporadic infantile epileptic encephalopathy caused by mutations in PCDH19 resembles Dravet syndrome but mainly affects females. PLoS Genet.

[6] Terracciano A, Trivisano M, Cusmai R, De Palma L, Fusco L, Compagnucci C (2016). PCDH19-related epilepsy in two mosaic male patients. Epilepsia.

[7] Hoshina N, Johnson-Venkatesh EM, Hoshina M, Umemori H (2021). Female-specific synaptic dysfunction and cognitive impairment in a mouse model of PCDH19 disorder. Science.

[8] Pederick DT, Richards KL, Piltz SG, Kumar R, Mincheva-Tasheva S, Mandelstam SA (2018). Abnormal cell sorting underlies the unique X-linked inheritance of PCDH19 epilepsy. Neuron.

[9] Emond MR, Biswas S, Blevins CJ, Jontes JD (2011). A complex of Protocadherin-19 and N-cadherin mediates a novel mechanism of cell adhesion. J Cell Biol.

[10] Bassani S, Cwetsch AW, Gerosa L, Serratto GM, Folci A, Hall IF (2018). The female epilepsy protein PCDH19 is a new GABAAR-binding partner that regulates GABAergic transmission as well as migration and morphological maturation of hippocampal neurons. Hum Mol Genet.

[11] Tai K, Kubota M, Shiono K, Tokutsu H, Suzuki ST (2010). Adhesion properties and retinofugal expression of chicken protocadherin-19. Brain Res.

[12] Cooper SR, Jontes JD, Sotomayor M (2016). Structural determinants of adhesion by Protocadherin-19 and implications for its role in epilepsy. eLife.

[13] Serratto GM, Pizzi E, Murru L, Mazzoleni S, Pelucchi S, Marcello E (2020). The epilepsy-related protein PCDH19 regulates tonic inhibition, GABAAR kinetics, and the intrinsic excitability of hippocampal neurons. Mol Neurobiol.

[14] Pham DH, Tan CC, Homan CC, Kolc KL, Corbett MA, McAninch D (2017). Protocadherin 19 (PCDH19) interacts with paraspeckle protein NONO to co-regulate gene expression with estrogen receptor alpha (ERalpha). Hum Mol Genet.

[15] Gerosa L, Mazzoleni S, Rusconi F, Longaretti A, Lewerissa E, Pelucchi S (2022). The epilepsy-associated protein PCDH19 undergoes NMDA receptor-dependent proteolytic cleavage and regulates the expression of immediate-early genes. Cell Rep.

[16] Biagini G, Panuccio G, Avoli M (2010). Neurosteroids and Epilepsy. Curr Opin Neurol.

[17] Tan C, Shard C, Ranieri E, Hynes K, Pham DH, Leach D (2015). Mutations of protocadherin 19 in female epilepsy (PCDH19-FE) lead to allopregnanolone deficiency. Hum Mol Genet.

[18] Trivisano M, Lucchi C, Rustichelli C, Terracciano A, Cusmai R, Ubertini GM (2017). Reduced steroidogenesis in patients with PCDH19-female limited epilepsy. Epilepsia.

[19] Lappalainen J, Chez M, Sullivan JE, Gecz J, Specchio N, Patroneva A (2017). A multicenter, open-label trial of ganaxolone in children with PCDH19 epilepsy (P5.236). Neurology.

[20] Sullivan J, Gunning B, Zafar M, Guerrini R, Gecz J, Kolc KL (2023). Phase 2, placebo-controlled clinical study of oral ganaxolone in PCDH19-clustering epilepsy. Epilepsy Res.

[21] Ueno K, Hirata H, Hinoda Y, Dahiya R (2013). Frizzled homolog proteins, microRNAs and Wnt Signaling in cancer. Int J Cancer.

[22] Honda A, Usui H, Sakimura K, Igarashi M (2017). Rufy3 is an adapter protein for small GTPases that activates a Rac guanine nucleotide exchange factor to control neuronal polarity. JBC.

[23] Dindler Rasmussen K, Helin K (2016). Role of TET enzymes in DNA methylation, development, and cancer. Genes Dev.

[24] Davey RA, Grossmann M (2016). Androgen receptor structure, function and biology: from bench to bedside. Clin Biochem Rev.

[25] Aquino EM, Benton MC, Haupt LM, Sutherland HG, Griffiths LR. Current understanding of DNA methylation and age-related disease. OBM Genet. 2018;2:2.

[26] Troung TH, Lange CA (2018). Deciphering steroid receptor crosstalk in hormone-driven cancers. Endocrinology.

[27] Zhang Q, Liu W, Zhang H-M, Xie G-Y, Miao Y-R, Xia M (2020). hTFtarget: A comprehensive database for regulations of human transcription factors and their targets. Genom. Proteom Bioinforma.

[28] Fu X, Pereira R, De Angelis C, Veeraraghavan J, Nanda S, Qin L (2019). FOXA1 upregulation promotes enhancer and transcriptional reprogramming in endocrine-resistant breast cancer. PNAS.

[29] Hurtado A, Holmes KA, Ross-Innes CS, Schmidt D, Carroll JS (2011). FOXA1 is a critical determinant of Estrogen receptor function and endocrine response. Nat Genet.

[30] Pham HD, Pitman MR, Kumar R, Jolly LA, Schulz R, Gardner AE (2021). Integrated in silico and experimental assessment of disease relevance of PCDH19 missense variants. Hum Mutat.

[31] Kahr I, Vandepoele K, van Roy F (2013). Delta-protocadherins in health and disease. Prog Mol Biol Transl Sci.

[32] Shen L, Inoue A, He J, Liu Y, Lu F, Zhang Y (2014). Tet3 and DNA replication mediate demethylation of both the maternal and paternal genomes in mouse zygotes. Cell Stem Cell.

[33] Kremer EA, Gaur N, Lee MA, Engmann O, Bohacek J, Mansuy IM (2018). Interplay between TETs and microRNAs in the adult brain for memory formation. Sci Rep.

[34] Yu H, Su Y, Shin J, Zhong C, Guo JU, Weng Y-L (2015). Tet3 regulates synaptic transmission and homeostatic plasticity via DNA oxidation and repair. Nat Neurosci.

[35] Homan CC, Pederson S, To T-H, Tan C, Piltz S, Corbett MA (2018). PCDH19 regulation of neural progenitor cell differentiation suggests asynchrony of neurogenesis as a mechanism contributing to PCDH19 girls clustering epilepsy. Neurobiol Dis.

[36] Panet-Raymond V, Gottlieb B, Beitel LK, Pinsky L, Trifiro MA (2000). Interactions between androgen and estrogen receptors and the effects on their transactivational properties. Mol Cell Endocrinol.

[37] McAbee MD, DonCarlos LL (1999). Estrogen, but not androgens, regulates androgen receptor messenger ribonucleic acid expression in the developing male rat forebrain. Endocrinology.

[38] Delić D, Grosser C, Dkhil M, Al-Quraishy S, Wunderlich F (2010). Testosterone-induced upregulation of miRNAs in the female mouse liver. Steroids.

[39] Gegenhuber B, Wu MV, Bronstein R, Tollkuhn J (2022). Gene regulation by gonadal hormone receptors underlies brain sex differences. Nature.

[40] Arambula SE (2017). Prenatal bisphenol A (BPA) exposure alters the transcriptome of the neonate rat amygdala in a sex-specific manner: a CLARITY-BPA consortium study. Neurotoxicology.

[41] Englert NA, Spink BC, Spink DC (2010). Persistent and non-persistent changes in gene expression result from long-term estrogen exposure of MCF-7 breast cancer cells. J Steroid Biochem Mol Biol.

[42] Velíšková J, De Jesus G, Kaur R, Velíšek L (2010). Females, their estrogens and seizures. Epilepsia.

[43] Reddy DS (2017). The neuroendocrine basis of sex differences in epilepsy. Pharm Biochem Behav.

[44] Velisek L, Nebieridze N, Chachua T, Veliskova J (2013). Anti-seizure medications and estradiol for neuroprotection in epilepsy: the 2013 update. Recent Pat CNS Drug Discov.

[45] Pederick DT, Homan CC, Jaehne EJ, Piltz SG, Haines BP, Baune BT (2016). Pcdh19 loss-of-function increases neuronal migration in vitro but is dispensable for brain development in mice. Sci Rep.

[46] Patro R, Duggal G, Love MI, Irizarry RA, Kingsford C (2017). Salmon provides fast and bias-aware quantification of transcript expression. Nat Methods.

[47] Ge SX, Jung D, Yao R (2019). ShinyGO: a graphical gene-set enrichment tool for animals and plants. Bioinformatics.

[48] Kaech S, Banker G (2007). Culturing hippocampal neurons. Nat Protoc.

[49] Laemmli UK (1970). Cleavage of structural proteins during the assembly of the head of bacteriophage T4. Nature.

[50] Hodgkinson K, Forrest LA, Nhung Vuong N, Garson K, Djordjevic B, Vanderhyden BC (2018). GREB1 is an estrogen receptor-regulated tumour promoter that is frequently expressed in ovarian cancer. Oncogene.

[51] Jia L, Kim J, Shen H, Clark PE, Tilley WD, Coetzee GA (2003). Androgen receptor activity at the prostate specific antigen locus: steroidal and non-steroidal mechanisms. Mol Cancer Res.

[52] Martinez CA, Marteinsdottir I, Josefsson A, Sydsjö G, Theodorsson E, Rodriguez-Martinez H (2020). Expression of stress-mediating genes is increased in term placentas of women with chronic self-perceived anxiety and depression. Genes.

