# Supplementary material for: Multiomic analysis implicates nuclear hormone receptor signalling in clustering epilepsy
Source: Transl Psychiatry. 2024 Jan 27;14:65. doi: 10.1038/s41398-024-02783-5 (PMC10821879; doi:10.1038/s41398-024-02783-5)
Supplement: Supplementary file 1 — Supplementary Information [file 41398_2024_2783_MOESM1_ESM.pdf]

Supplementary Information:

Multiomic analysis implicates Nuclear Hormone Receptor signalling in Clustering Epilepsy

Rebekah de Nys[1], Clare L. van Eyk[1], Tarin Ritchie[1], Rikke S. Møller[2,3], Ingrid E. Scheffer[4,5,6,7], Carla Marini[8], Rudrarup Bhattacharjee[1], Raman Kumar[1,†] and Jozef Gecz[1,9,†,*]

[1] Adelaide Medical School and Robinson Research Institute, The University of Adelaide, Adelaide, SA 5005, Australia.

[2] Department of Epilepsy Genetics and Personalized Medicine (member of ERN EpiCARE), Danish Epilepsy Centre, Filadelfia, Dianalund, Denmark

[3] Department of Regional Health Research, University of Southern Denmark, Odense, Denmark

[4] Epilepsy Research Centre, University of Melbourne, Austin Health, Heidelberg, Victoria, 3084, Australia.

[5] Department of Paediatrics, University of Melbourne, Parkville, Victoria, 3052, Australia.

[6] Department of Neurology, The Royal Children's Hospital, Parkville, Victoria, 3052, Australia.

[7] Murdoch Children's Research Institute, Parkville, Victoria, 3052, Australia.

[8] Child Neurology and Psychiatry Unit Children's Hospital "G. Salesi" Azienda Ospedaliero-Universitaria delle Marche Ancona, Ancona, Italy.

[9] South Australian Health and Medical Research Institute, Adelaide, SA 5000, Australia.

Supplementary Figures

Supplementary Fig. 1 Volcano plot of upregulated and downregulated DEGs for **(A)** AFs vs FCs and **(B)** TMs vs MCs.

A

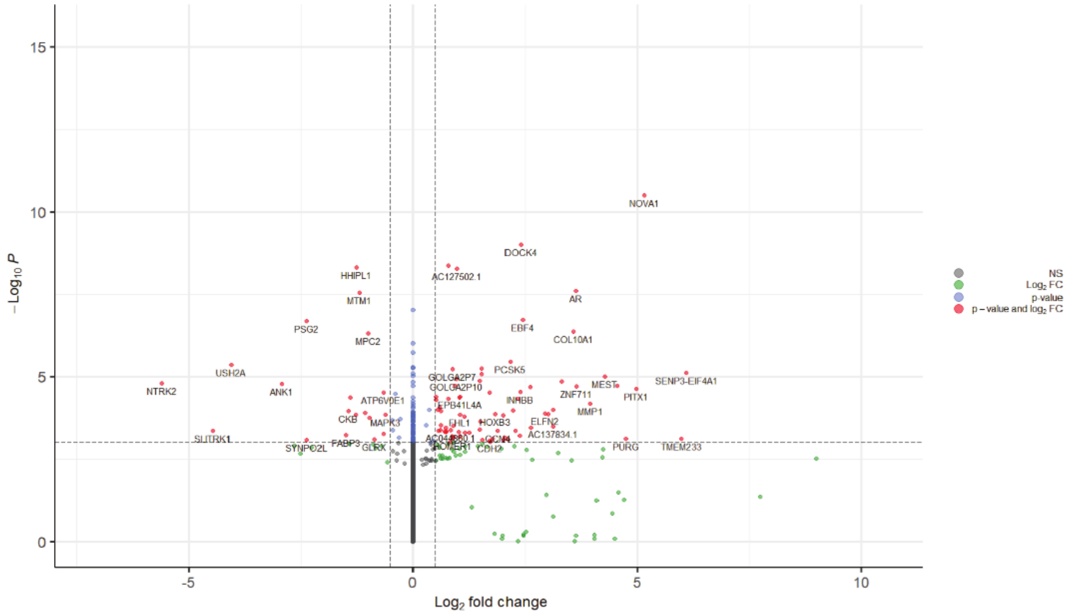

B

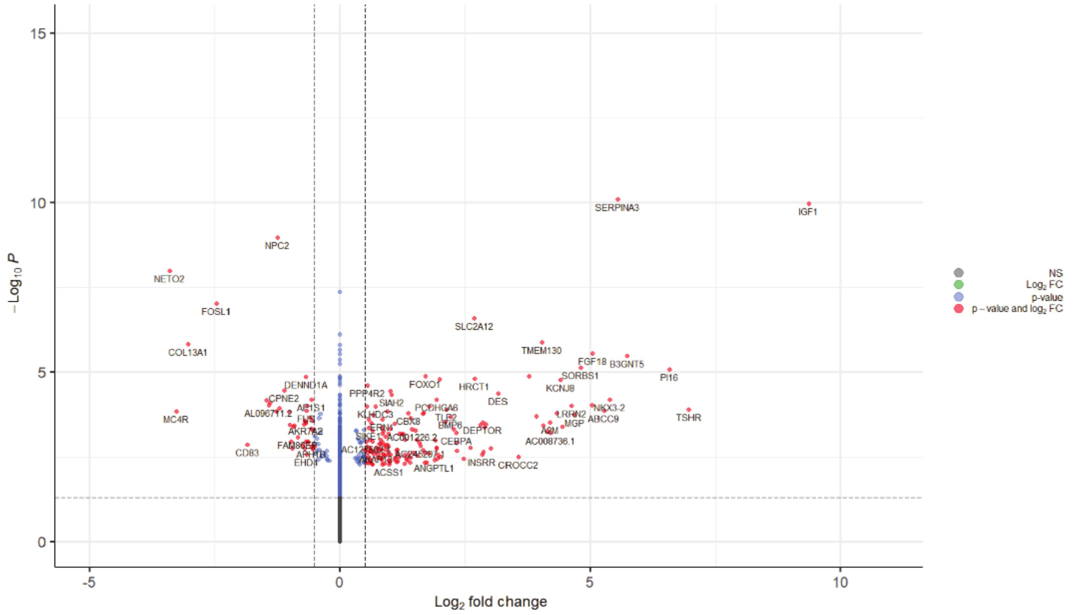

Supplementary Fig. 2 Comparison of the expression and methylation of selected genes in AFs and FCs. **(A)** Venn diagram of the number of genes with both significant differential expression (DEG) and methylation of the promoter or gene body (DMR) in AFs when compared to FCs. Comparison of expression and methylation of **(B)** *RUFY3*, **(C)** *HOXB3*, **(D)** *SERPINA3*, **(E)** *LHX8* and **(F)** *TET3* in AFs and FCs.

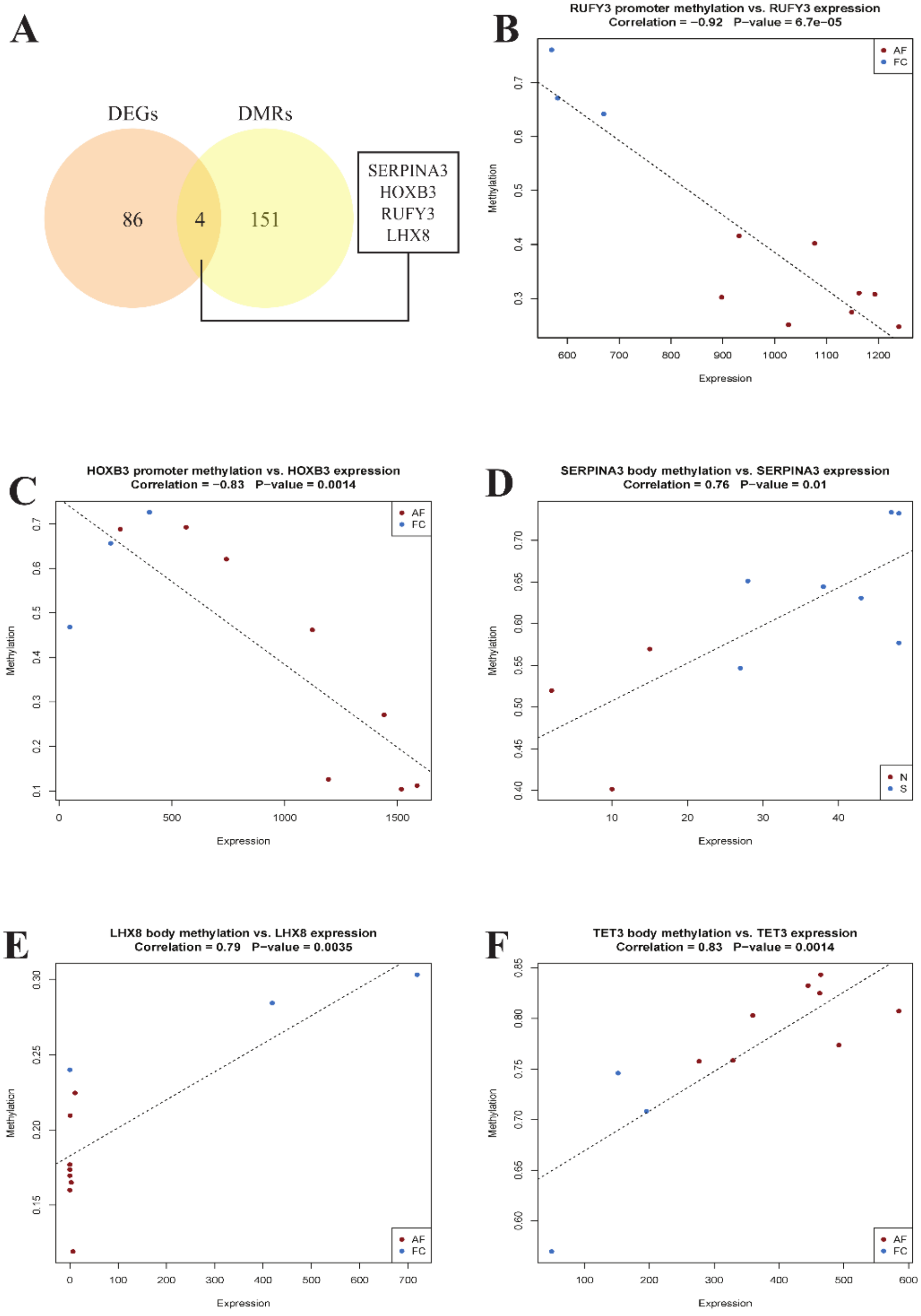

Supplementary Fig. 3 Western blot of T47D, ZR-75-1 and MCF-7 breast cancer lysate was probed with anti-AR, anti-PGR, anti-ERα and anti-β-Tubulin (loading control) antibodies.

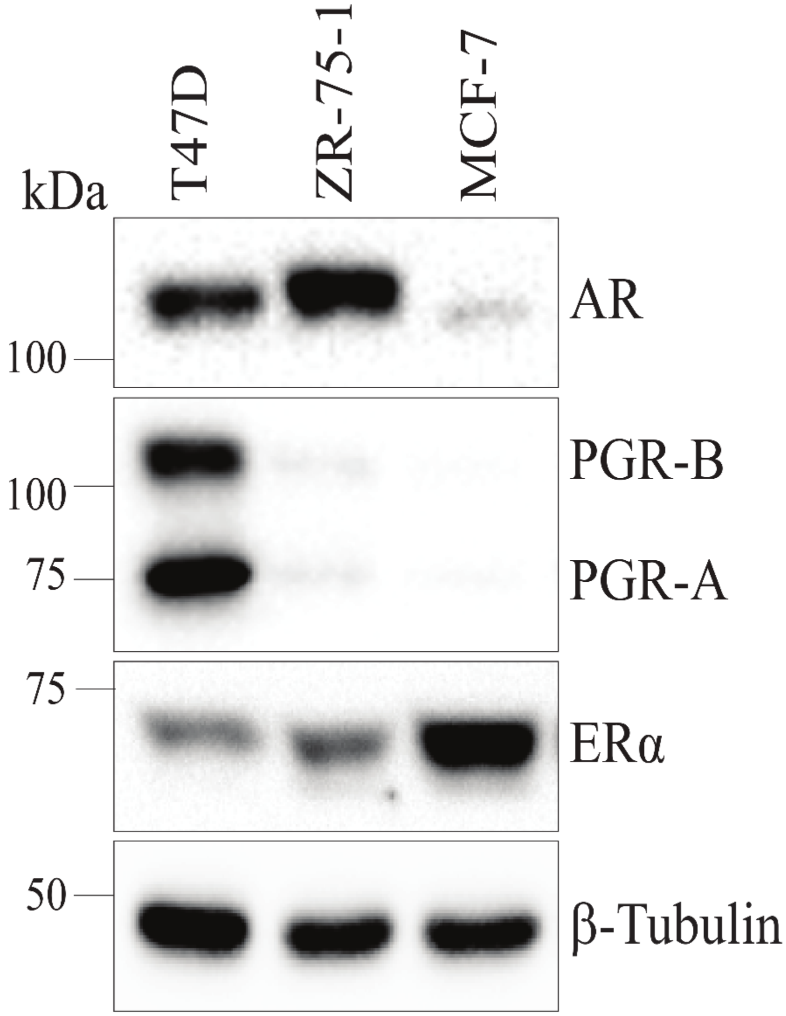

Supplementary Fig. 4 Western blot of T47D cells treated with steroids for 4, 6, 16, 24 and 48 hours. Cells treated with **(A)** 10 nM E2, **(B)** 10 nM P4, **(C)** 10 nM DHT or vehicle (-). Protein lysates were western blotted and probed with anti-ERα, anti-PGR, anti-AR or anti-β-Tubulin (loading control) antibodies.

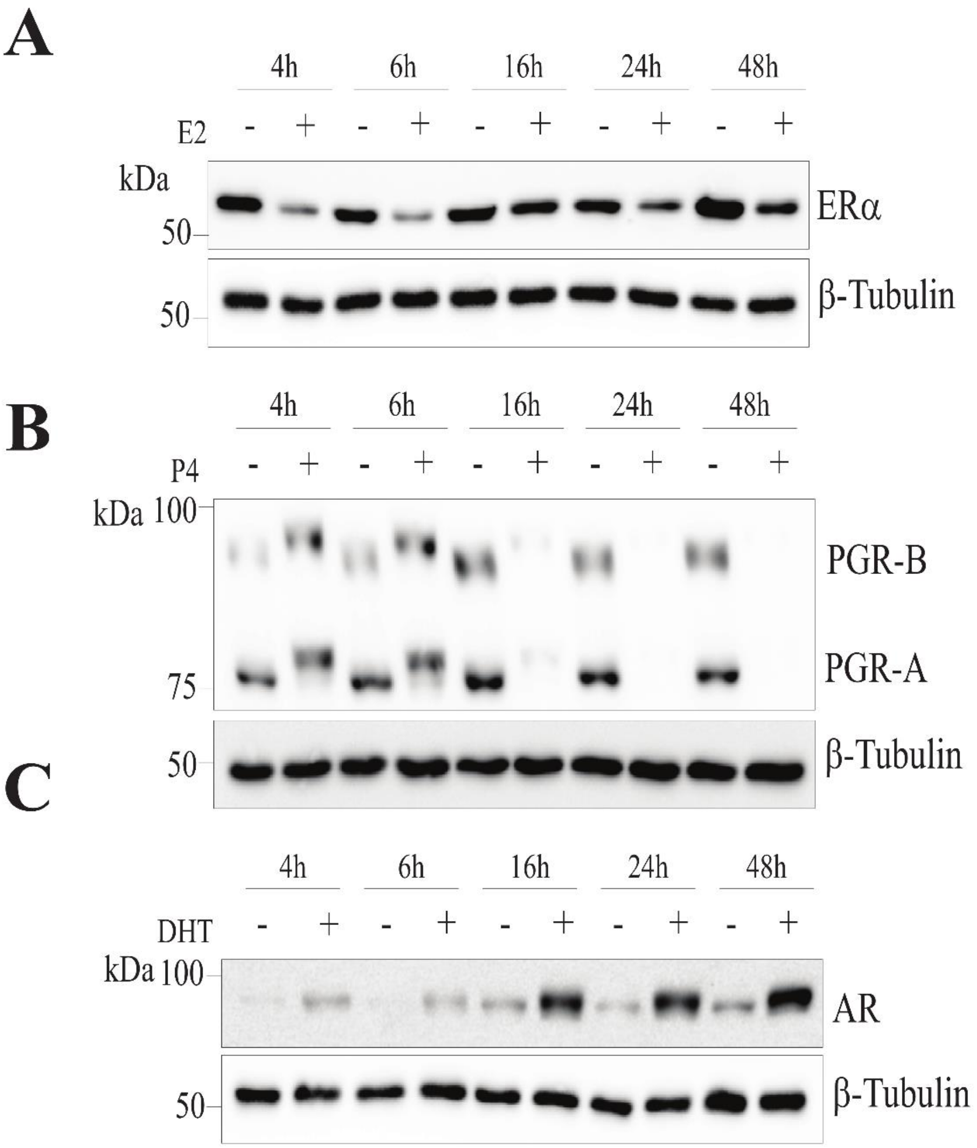

Supplementary Fig. 5 Expression of known NHR target genes after steroid treatment in T47D cells. Expression of known **(A)** ERα target genes (*TFF1* and *GREB1*, n=3), **(B)** PGR target genes (*HSD11B2* and *PPL*, n=3) and **(C)** AR target genes (*PIP* and *CDKN1A*, n≥2) were assessed in cells treated with their respective ligands by RT-qPCR. Statistical analysis was performed using one-way ANOVA for each timepoint. *=p (≤0.05), ***= p (≤0.001), ****= p (≤0.0001).

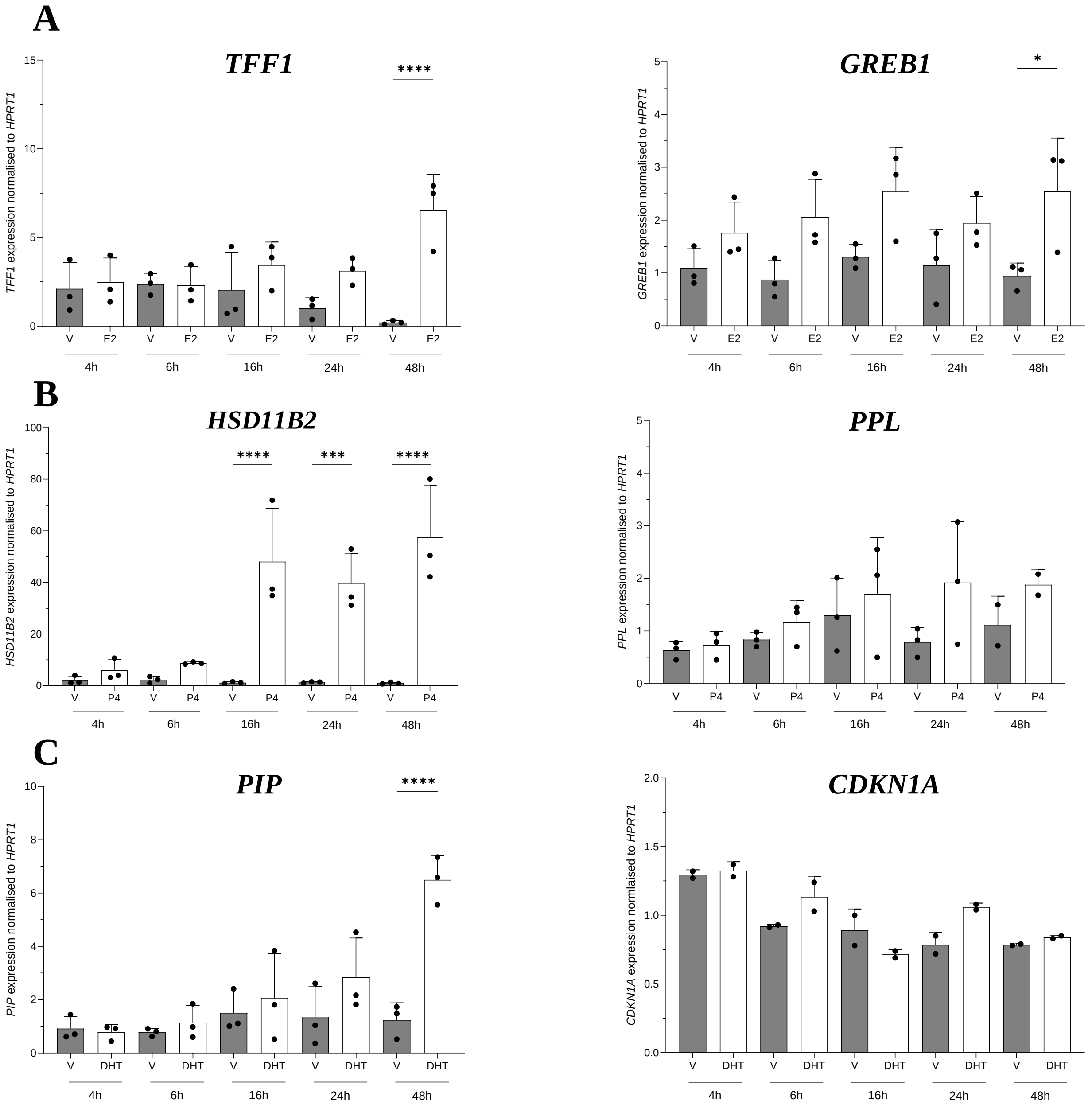

Supplementary Fig. 6 *PCDH19* mRNA expression in T47D cells cultured in non-stripped (RPMI+phenol red+10% FBS) or stripped (RPMI-phenol red+10% DCC-FBS) media for the time indicated. n=3. Statistical analysis performed using one-way ANOVA. ***= p (≤0.001), ****=p (≤0.0001).

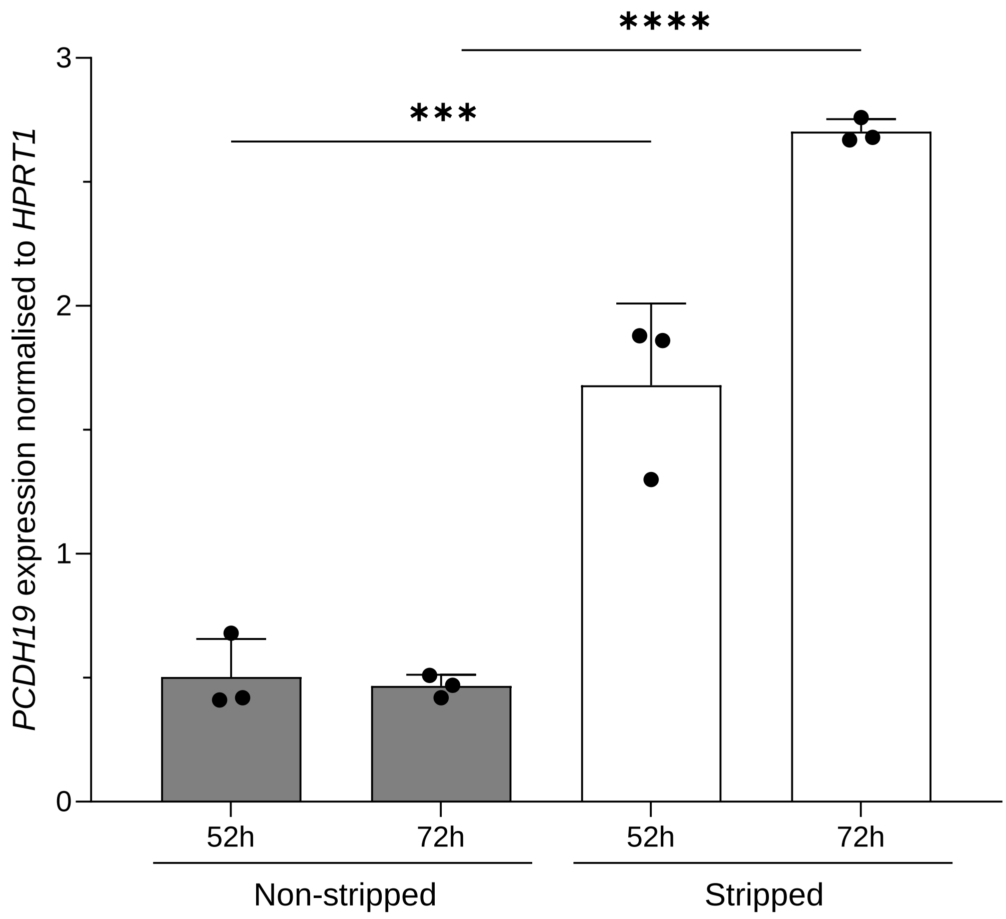

Supplementary Fig. 7 Expression of known NHR target genes after steroid treatment in female E18.5 mouse cortical neurons. Expression of known **(A)** ERα target genes (*Rara* and *Nrip1*, n=3), **(B)** PGR target genes (*Hsd11b2* and *Ppl*, n=3) and **(C)** AR target genes (*Cdkn1a* and *Pmepa1*, n=3) were assessed in neurons treated with their respective ligands by RT-qPCR. Statistical analysis was performed using unpaired t-test. **= p (≤0.01).

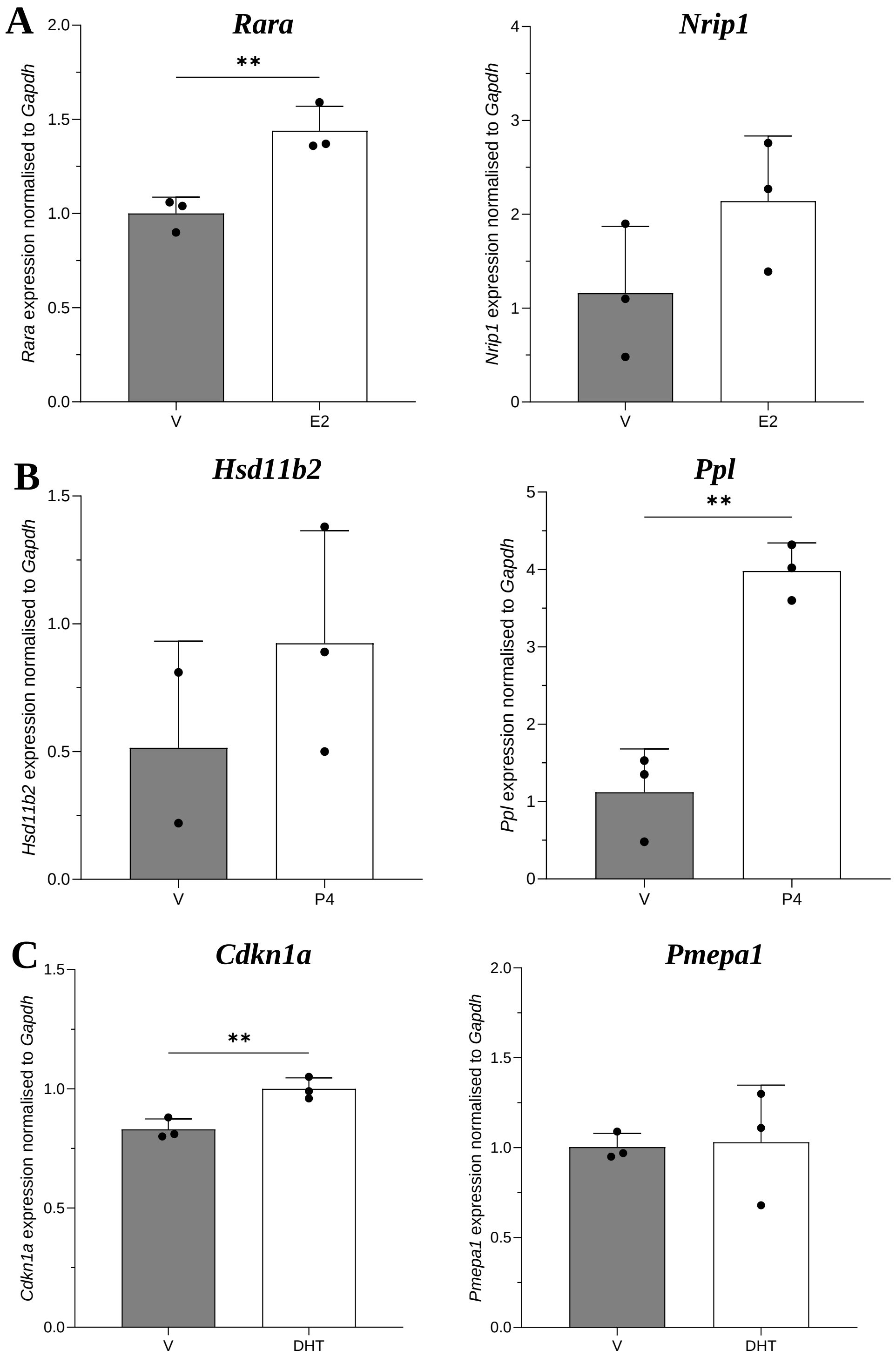

Supplementary Fig. 8 Expression of known NHR target genes after steroid treatment in male E18.5 mouse cortical neurons. Expression of known **(A)** ERα target genes (*Rara* and *Nrip1*, n=3), **(B)** PGR target genes (*Hsd11b2* and *Ppl*, n=3) and **(C)** AR target genes (*Pip* and *Cdkn1a*, n≥2) were assessed in neurons treated with their respective ligands by RT-qPCR. Statistical analysis was performed using unpaired t-test. **= p (≤0.01).

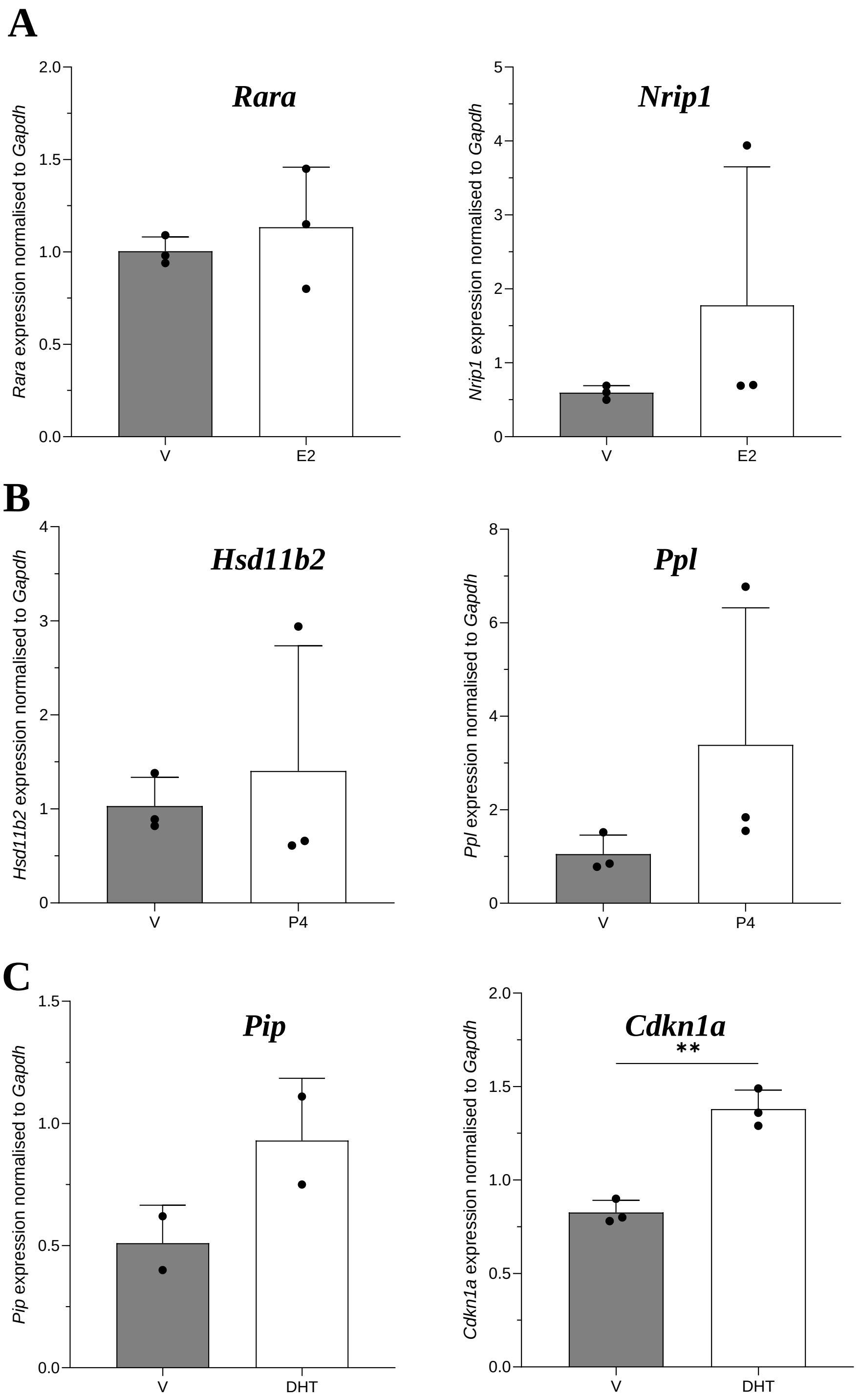

Supplementary Fig. 9 T47D cells were treated with vehicle or 10 nM E2, P4, DHT, E2+P4, E2+DHT, P4+DHT, E2+P4+DHT for 24 hours. *PCDH19* mRNA expression was determined by RT-qPCR and normalised to *HPRT1* expression. n=3. Statistical analysis performed using one-way ANOVA. *=p (≤0.05), **= p (≤0.01).

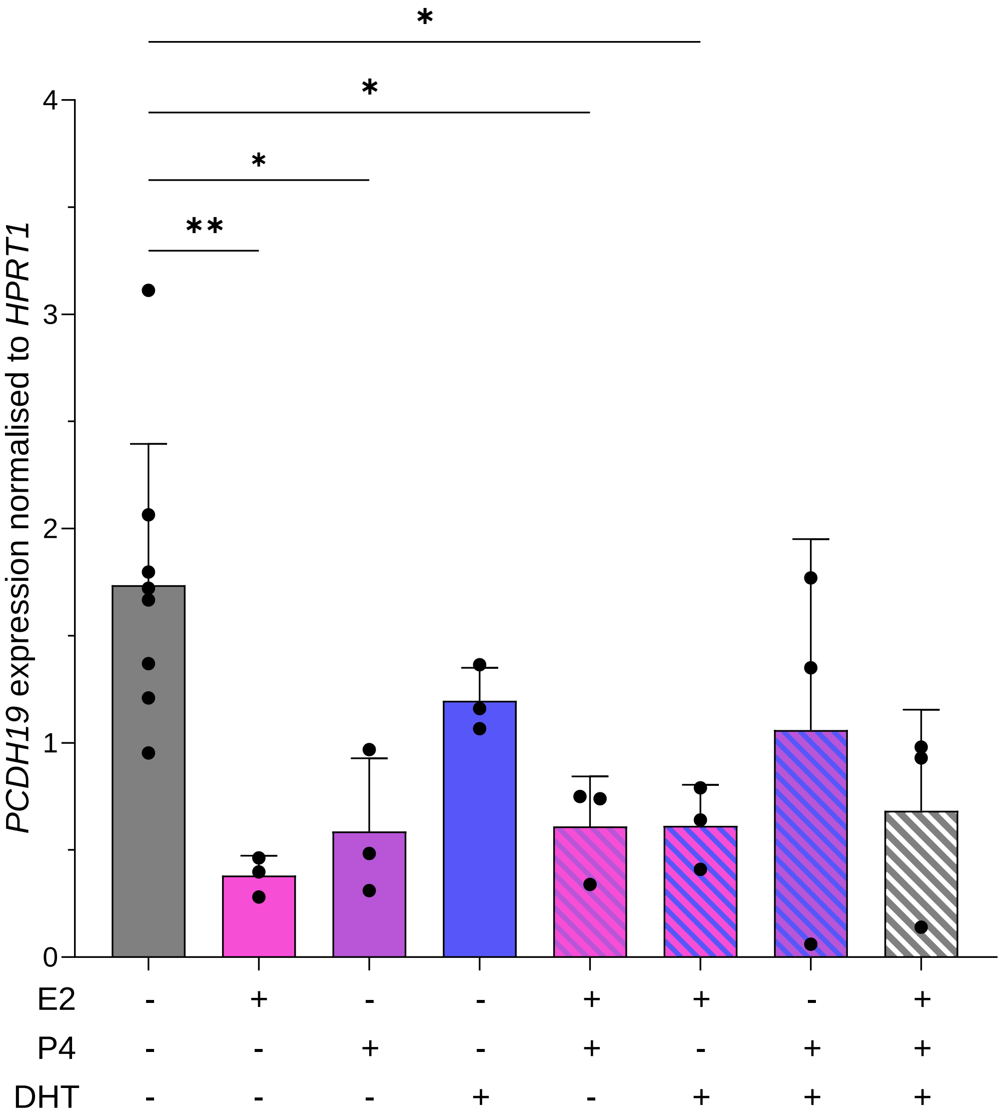

Supplementary Fig. 10 ERα, PGR and AR were knocked down in T47D cells using two siRNA molecules each. Successful knockdown was confirmed by western blotting.

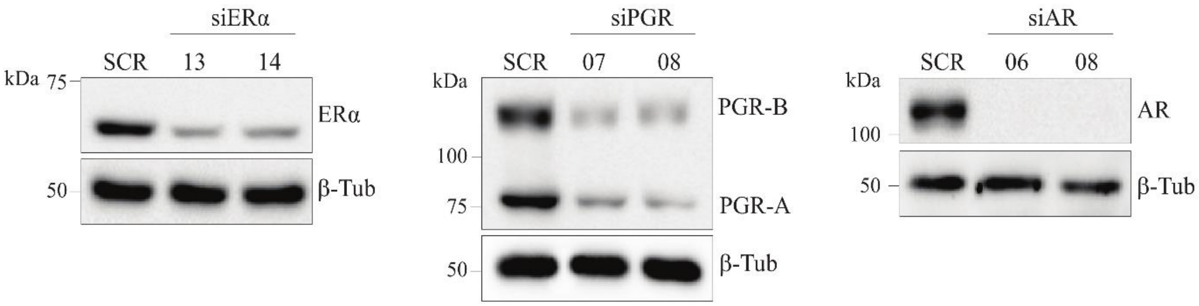

Supplementary Fig. 11 *PCDH19* mRNA expression in T47D cells treated with increasing E2 concentrations for 24 hours. n=2.

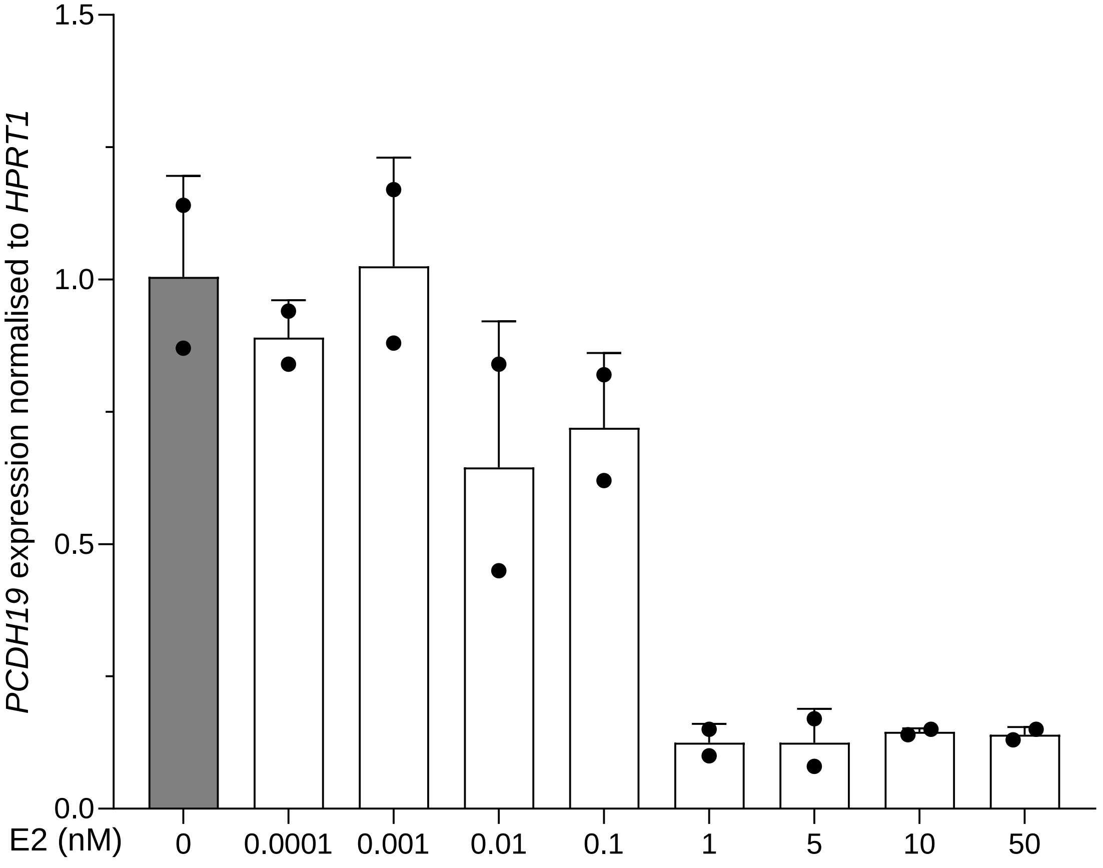

Supplementary Fig. 12 FOXA1 was knocked down in T47D cells using two siRNA molecules and treated with E2 for 24 hours. Successful knockdown was confirmed by western blotting.

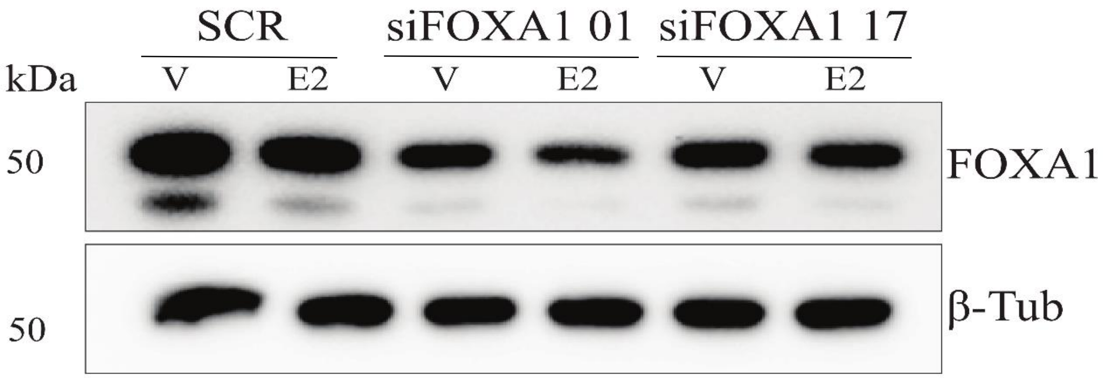

Supplementary Fig. 13 FOXA1 was knocked down in T47D cells using two siRNA molecules and treated with E2 for 24 hours. *TFF1* mRNA expressing was determined by RT-qPCR. N=3.Statistical analysis was performed using one-way ANOVA. ****=p (≤0.0001).

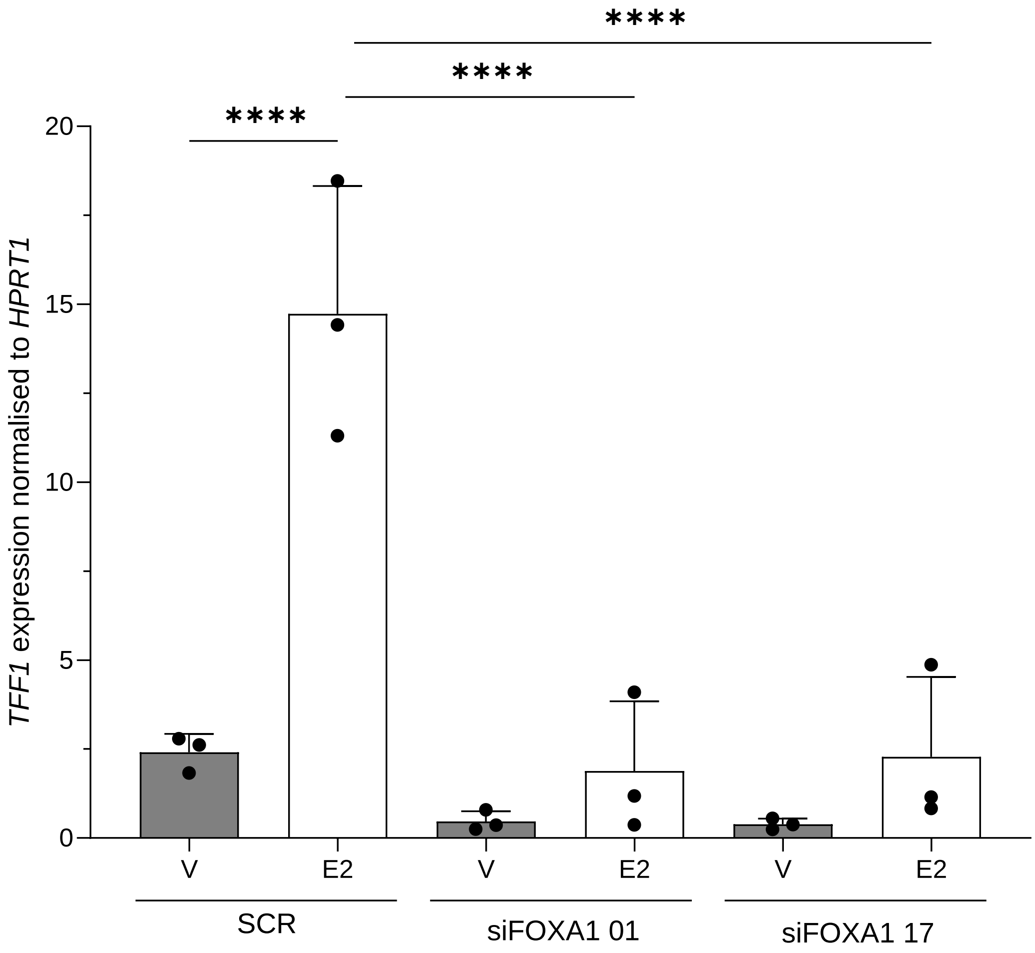

Supplementary Tables

Supplementary Table 1- Patient clinical information. Provided as a separate file.

Supplementary Table 2- CE skin fibroblasts DMPs. Provided as a separate file.

Supplementary Table 3- CE skin fibroblasts DMRs. Provided as a separate file.

Supplementary Table 4- CE skin fibroblasts DEGs. Provided as a separate file.

Supplementary Table 5- Background list used for GO enrichment analysis. Provided as a separate file.

Supplementary Table 6. List of Taqman probes.

Taqman Probe	Company	Cat #
AR	Thermo Fisher	Hs00171171_m1
CDKN1A	Thermo Fisher	Hs00355782_m1
Cdkn1a	Thermo Fisher	Mm04205640_g1
ESR1	Thermo Fisher	Hs01046816_m1
Gapdh	Thermo Fisher	Mm99999915_g1
HPRT1	Life Technologies	4325801
Hsd11b2	Thermo Fisher	Mm01251104_m1
NOVA1	Thermo Fisher	Hs00246362_m1
Nrip1	Thermo Fisher	Mm00476537_m1
PCDH19	Thermo Fisher	Hs00403382_m1
Pcdh19	Thermo Fisher	Mm01340474_m1
PGR	Thermo Fisher	Hs01556702_m1
Pip	Thermo Fisher	Mm00476800_m1
Pmepa1	Thermo Fisher	Mm00452230_g1
PPL	Thermo Fisher	Hs01011417_m1
Ppl	Thermo Fisher	Mm00447206_m1
Rara	Thermo Fisher	Mm01296312_m1
RUFY3	Thermo Fisher	Hs01127885_m1
TET3	Thermo Fisher	Hs00896441_m1
TFF1	Thermo Fisher	Hs00907239_m1
Tff1	Thermo Fisher	Mm00436945_m1

Supplementary Table 7. List of primers used for RT-qPCR.

Target	Primer	Sequence 5'-3'	Reference
GREB1	F	TAGCGACCCCTGGCCAGACC	[50]
	R	GCCGTCTGACGCCGCACATA	
HPRT1	F	TGACACTGGCAAAACAATGCA	[17]

	R	GGTCCTTTTCACCAGCAAGCT	
HSD11B2	F	TGCTTCAAGACAGAGTCAGTG	52
	R	GGCATCTACAACTGGGGTGA	
PSA	F	GGCAGCATTGAACCAGAGGAG	51
	R	GCATGAACTTGGTCACCTTCTG	

Supplementary Table 8. List of primers used for cloning the *PCDH19* regulatory fragment with FOXA1 binding sites into pGL2-TK2 and FOXA1 binding site mutagenesis.

**Name**	**Primer**	**Sequence 5'-3'**
PCDH 19 promoter	F	AGCTAACATAACCCGGGCTTCCACTACCAGA GAATGAGAAGAGA
	R	GGCCGGATCTCGAGGAGCTGTGCTGCCGTCTGTGCCCGCT
BS1+BS2 Mutagenesis	F	TATGAAGATATGTAAGGGAGGGACCCGATAAGACAAT CATATCATCCTCATCTTTCCTGAAATCTCTGCACCTGA GAAGCGCACAG
	R	CTGTGCGCTTCTCAGGTGCAGAGATTTCAGGAAAGATGAGG ATGATATGATTGTCTTATCGGGTCCCTCCCTTACATATCTTC ATA
BS1 Mutagenesis	F	TATGAAGATATGTAAGGGAGGGACCCGATAAGACAA
	R	TTGTCTTATCGGGTCCCTCCCTTACATATCTTCATA
BS2 Mutagenesis	F	TTTCCTGAAATCTCTGCACCTGAGAAGCGCACAG
	R	CTGTGCGCTTCTCAGGTGCAGAGATTTCAGGAAA
